# Sirt3 Genetically Engineered Apoptotic Bodies Alleviate Skeletal Aging by Limiting Aggravated NLRP3 Inflammasome Activation of Senescent Macrophages

**DOI:** 10.1002/advs.202517554

**Published:** 2026-02-27

**Authors:** Yanglin Wu, Shifeng Ling, Jiayi Mao, Hongyi Wang, Bo Wang, Zhenjia Che, Wenguo Cui, Ming Cai

**Affiliations:** ^1^ Department of Orthopaedics Shanghai Tenth People's Hospital School of Medicine, Tongji University Shanghai P. R. China; ^2^ Department of Orthopaedics Shanghai Key Laboratory for Prevention and Treatment of Bone and Joint Diseases Shanghai Institute of Traumatology and Orthopaedics Ruijin Hospital Shanghai Jiao Tong University School of Medicine Shanghai P. R. China; ^3^ Department of Plastic and Reconstructive Surgery Shanghai Ninth People's Hospital Shanghai Jiao Tong University School of Medicine Shanghai P. R. China; ^4^ Department of Spinal Surgery Shanghai East Hospital School of Medicine Tongji University Shanghai P. R. China

**Keywords:** apoptotic body, macrophage, NLRP3 inflammasome, skeletal aging

## Abstract

Skeletal aging is characterized by increased fragility, reduced bone mass, and deterioration of bone microstructure. Although aging‐related immune dysfunction of macrophages, namely immunosenescence, is known to contribute to this process, the underlying mechanism remains poorly understood. Here, we find that the senescence of macrophages leads to a decrease in the expression of Sirtuin3 (Sirt3), which in turn leads to increased basal and lipopolysaccharides (LPS)‐induced protein expression of NLRP3 and facilitates the assembly of NLRP3 inflammasome in macrophages that mediates aging‐related osteoporosis. Given the phagocytic property of macrophages, we develop a genetically engineered apoptotic body‐based platform for targeted delivery of Sirt3 to macrophages and verify that Sirt3‐enriched apoptotic bodies (ABs‐Sirt3) delay skeletal aging by promoting ubiquitination and degradation of NLRP3. Our work reveals that Sirt3 plays a key role in regulating aggravated inflammatory responses that accelerate skeletal aging during macrophage senescence and illustrates a novel nanotechnology‐based therapeutic approach targeting immune senescence‐induced acceleration of skeletal aging, which may provide potential therapeutic value for human patients with age‐related osteoporosis.

## Graphical abstract

The senescence of macrophages leads to a decrease in the expression of Sirtuin3 (Sirt3), which in turn aggravates the NLRP3 inflammasome activation and IL‐1β secretion, thereby promoting the aging‐related osteoporosis. Sirt3‐enriched apoptotic bodies (ABs‐Sirt3) generated through genetic editing approaches can alleviate skeletal aging by targeting macrophages for the delivery of Sirt3.

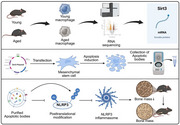

## 1 Introduction

Age‐related osteoporosis, characterized by skeletal disorders, is increasingly prevalent due to global population aging. Old people who are suffering from osteoporosis are prone to bone fractures, which is undoubtedly a heavy economic burden to society and may reduce the quality of life [1]. Although drugs that promote osteogenesis and/or inhibit osteoclastogenesis have been improved to obtain better therapeutic effects, age‐related osteoporosis is still a great challenge. Previous studies have shown that systemic low‐grade inflammation driven by the process of aging will accelerate age‐related diseases, which terms inflamm‐aging [2]. Targeting inflamm‐aging may provide insights into managements of senile osteoporosis. As a unique part of innate immunity, the aberrant NLRP3 (Nod‐like receptor family protein 3) inflammasome activation is responsible for such kind of systemic low‐grade inflammation [3, 4, 5].

The NLRP3 inflammasome is composed mainly of a sensor NLRP3, an effector caspase‐1, and an adaptor ASC. Upon activated, NLRP3 inflammasome will assemble and lead to the auto‐maturation of caspase‐1, which in turn induces the cleavage of IL‐1β pro and IL‐18 pro. Structurally distinct DAMPs such as cholesterol crystals, amyloids, and MSU (monosodium urate) can be sensed by NLRP3, which mediates the pathogenesis of atherosclerosis, Alzheimer's disease, and gout [6, 7, 8]. Notably, bone metabolism has also been regulated by NLRP3 inflammasome [3, 9]. Meanwhile, the effects of aging on the NLRP3 inflammasome activation of macrophages are poorly understood.

The Sirtuins family, nicotinamide adenine dinucleotide‐dependent (NAD^+^) deacetylases, is regarded as a key delayer of aging according to recent studies [10, 11, 12, 13]. The family members Sirtuin1‐7 (Sirt1‐7) have been implied to regulate age‐related diseases, including aging‐induced vascular remodeling, osteoarthritis, insulin resistance, and so on [14, 15, 16, 17]. As a less characterized member of Sirtuins, Sirt3 receives more and more attention for its irreplaceable role in aging and degenerative diseases. Like its family members of Sirt4 and Sirt5, Sirt3 is located in mitochondria and acted as a mitochondrial deacetylase [18, 19]. Due to the central role of mitochondria in metabolism, Sirt3 is unsurprisingly associated with most aspects of aging.

In this work, we find that the senescence of macrophages leads to a decrease in Sirt3 expression, which exacerbates the activation of the NLRP3 inflammasome and thereby accelerates skeletal aging. Given that Sirt3 lacks of specific activators in clinical practice and the gene therapy still faces great challenges in application, we designed a novel targeted delivery strategy based on the phagocytic property of macrophages. Apoptotic bodies, as a special type of extracellular vesicles, participate in a range of physiological and pathological mechanisms, which attracts more and more attention in recent years and is regarded as a promising therapeutic platform for drug delivery [20]. More importantly, apoptotic bodies can be specifically recognized by macrophages through eat‐me signals [21]. Based on these reasons, Sirt3‐enriched apoptotic bodies (ABs‐Sirt3) are constructed. We find ABs‐Sirt3 suppresses the NLRP3 inflammasome activation by reducing the acetylation of NLRP3 and promoting its ubiquitin‐mediated degradation, which in turn alleviates skeletal aging accelerated by aggravated immune responses of senescent macrophages.

## 2 Results

### 2.1 Aging Facilitated the NLRP3 Inflammasome Activation of Macrophages and In Turn Induced Age‐Related Osteoporosis

**FIGURE 1:**
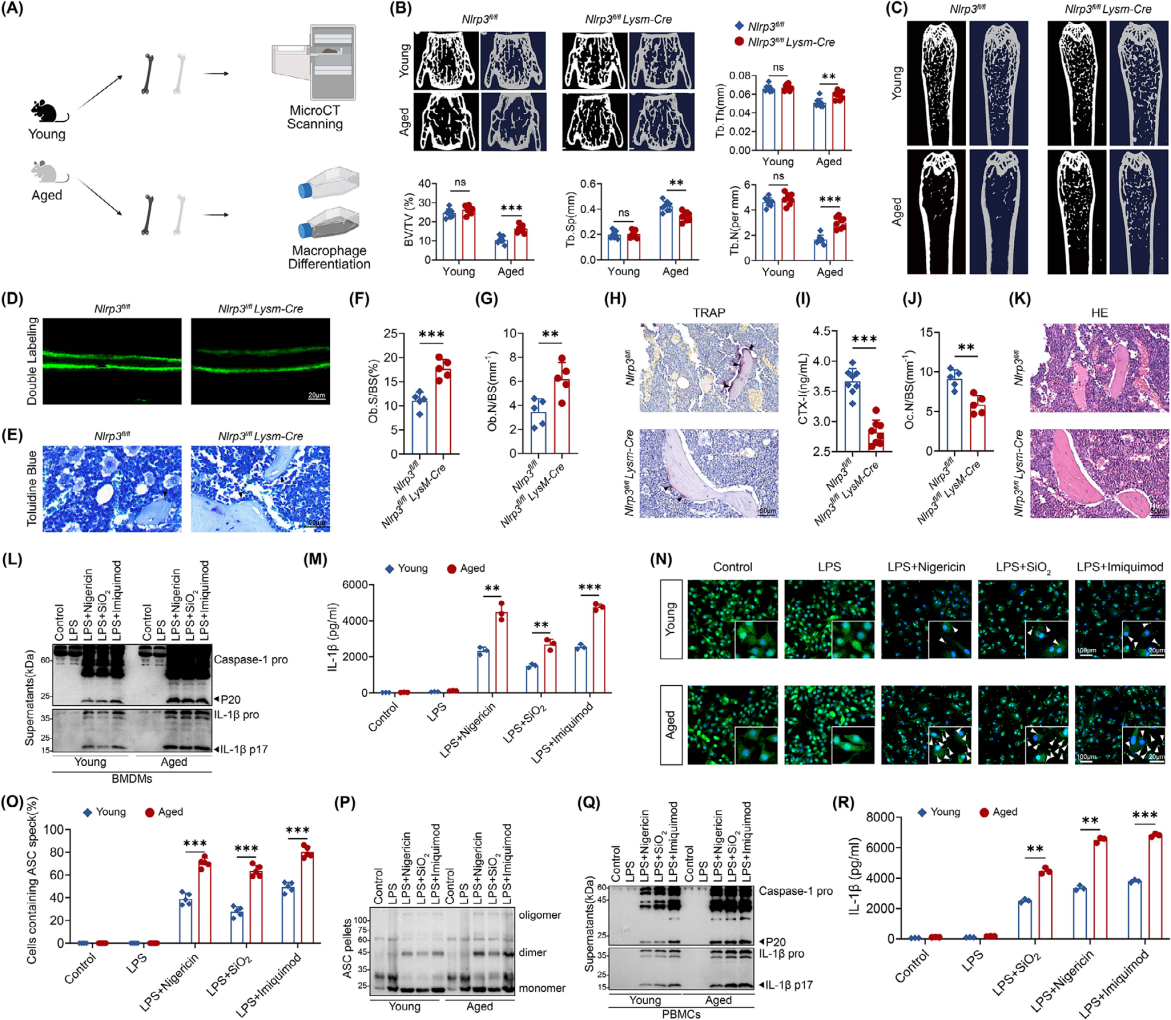
Deactivation of NLRP3 inflammasome alleviated aging‐related osteoporosis. (A) Schematic diagram of experiments investigating the role of senescent macrophages in immune response and bone mass regulation. (B) 3D‐reconstructed MicroCT images of vertebral and quantitative femoral trabecular parameters: BV/TV (%), bone volume to tissue volume; Tb.Sp (mm), trabecular separation; Tb.Th (mm), trabecular thickness; Tb.N (1/mm); trabecular bone number. (*n* = 8). (C) Representative femur micro‐CT images. (D) Fluorescence micrographs of calcein double‐labeled trabecular bone sections of femur. (E) Toluidine blue (TB) staining of femur sectuons. Black arrows indicate osteoblasts. (F) Histomorphometric assessment of osteoblast coverage on trabecular surfaces of femur (Ob.S/BS). (G) Histomorphometric assessment of osteoblasts number on trabecular surfaces of femur (N.Ob/BS) (*n* = 5). (H) TRAP staining of distal femur. Black arrows indicate osteoclasts. (I) Serum level of CTX‐I in *Nlrp3^fl/fl^* and *Nlrp3^fl/fl^ LysM‐Cre* mice (*n* = 8). (J) Histomorphometric assessment of osteoclasts number on trabecular surfaces of femur (N.Oc/BS) (*n* = 5). (K) HE staining of distal femur. (L,M) Following LPS priming, BMDMs were exposed to distinct NLRP3 inflammasome activators: nigericin, SiO2, or Imiquimod, respectively. Supernatants were subjected to the analysis of caspase‐1 and IL‐1β by western blot (L), and the secretion of IL‐1β by ELISA (M) (*n* = 3). (N,O) Representative immunofluorescence images (N) and quantification (O) of ASC speck in LPS‐primed BMDMs stimulated with nigericin, SiO_2_, or Imiquimod, respectively (*n* = 5). (P) Following LPS priming, BMDMs were exposed to distinct NLRP3 inflammasome activators: nigericin, SiO_2_, or Imiquimod, respectively. Cell lysates were cross‐linked and subjected for the analysis of ASC oligomerization by immunoblot. (Q,R) Following LPS priming, PBMCs were exposed to distinct NLRP3 inflammasome activators: nigericin, SiO_2_, or Imiquimod, respectively. Supernatants were subjected to the analysis of caspase‐1 and IL‐1β by western blot (Q), and the secretion of cytokine IL‐1β (R) (*n* = 3). Results are presented as mean values with standard deviation (mean ± SD). Each point in the scatter plots represented one individual. All experiments were conducted with three or more independent biological replicates per group. Significance was assessed by two‐tailed unpaired t‐test: ^**^ *p* < 0.01, ^***^ *p* < 0.001.

Evidence suggested that blocking NLRP3 inflammasome signaling prevented tissue aging systemically [3]. To confirm whether the immune dysfunction of senescent macrophages was involved in skeletal aging, *Nlrp3^fl/fl^LysM‐Cre* mice with deletion of *Nlrp3* in myeloid cells were generated by crossing *Nlrp3* floxed mice with *LysM‐Cre* mice. No marked differences of bone mass between young (4 months) *Nlrp3^fl/fl^LysM‐Cre* mice and their littermates *Nlrp3^fl/fl^* mice (Wild type, WT) were observed, while aged (24 months) *Nlrp3^fl/fl^LysM‐Cre* mice showed a delayed bone loss with a higher level of trabecular thickness, trabecular bone volume, and trabecular bone number (Figure 1A–C).

Next, multiple methods were used to analyze the bone metabolism of NLRP3 conditional knockout (*Nlrp3^fl/fl^LysM‐Cre*) mice. Calcein double labeling showed a profoundly increased bone formation in femur trabecular bones of NLRP3 conditional knockout mice compared with littermate WT (*Nlrp3^fl/fl^*) controls (Figure 1D; Figure S1A,B). The serum level of OCN (Osteocalcin) and the number of TB (toluidine blue) labeled osteoblasts were dramatically higher in *Nlrp3^fl/fl^LysM‐Cre* mice (Figure 1E–G; Figure S1C). Inversely, the number of TRAP^+^ (tartaric acidic phosphatase) osteoclast and serum level of bone resorption marker CTX‐I (carboxy‐terminal type I collagen) cross‐linkage were much lower in *Nlrp3^fl/fl^ LysM‐Cre* mice than those in WT mice (Figure 1H–J; Figure S1D). Furthermore, the accumulation of adipocytes in the bone marrow cavity exhibited a significant positive correlation with skeletal aging. With the progression of aging, bone marrow mesenchymal stem cells tend to differentiate more readily into adipocytes rather than osteoblasts. Thus, increased accumulation of adipocytes may serve as an indicator of accelerated aging in the skeletal system. More area and number of adipocytes could be observed in femurs of *Nlrp3^fl/fl^LysM‐Cre* mice (Figure 1K). Similar to *Nlrp3* conditional knockout mice, differences in bone mass between WT and *Nlrp3* KO mice were much more significant in the elderly stage and confirmed by multiple histological assessments (Figure S2). Collectively, our results confirmed ablation of *Nlrp3* could delay skeletal aging.

To determine the impacts of aging on immune responses, we stimulated bone marrow derived macrophages (BMDMs) from young (4 months) and aged (24 months) mice with different NLRP3 agonists, including Nigericin (K^+^ efflux dependent), Imiquimod (K^+^efflux independent), and SiO_2_ (lysosomal rupture dependent). More matured caspase‐1 (cleaved capsae‐1, P20) and IL‐1β (cleaved IL‐1β, P17) were generated in aged macrophages compared with young macrophages (Figure 1L; Figure S1E,F). Aggravated activation of NLPR3 inflammasome was also evidenced by the nearly double amount of cytokine release in senescent macrophages compared with young macrophages (Figure 1M; Figure S1G). Besides, ASC speck formation and oligomerization were facilitated in aged macrophages (Figure 1N–P). Furthermore, similar results that aging boosted the maturation of caspase‐1 and IL‐1β could also be observed in human PBMCs from young and aged healthy donors. We observed that more cleaved caspase‐1 (P20) and IL‐1β (P17) were generated in senescent PBMCs (Figure 1Q; Figure S1H,I). Elderly donor PBMCs exhibited enhanced cytokine release following NLRP3 agonist stimulation (Figure 1R; Figure S1J). These results indicated that aging was accompanied by increased susceptibilities of macrophages upon the NLRP3 inflammasome activation and induced skeletal aging.

**FIGURE 2:**
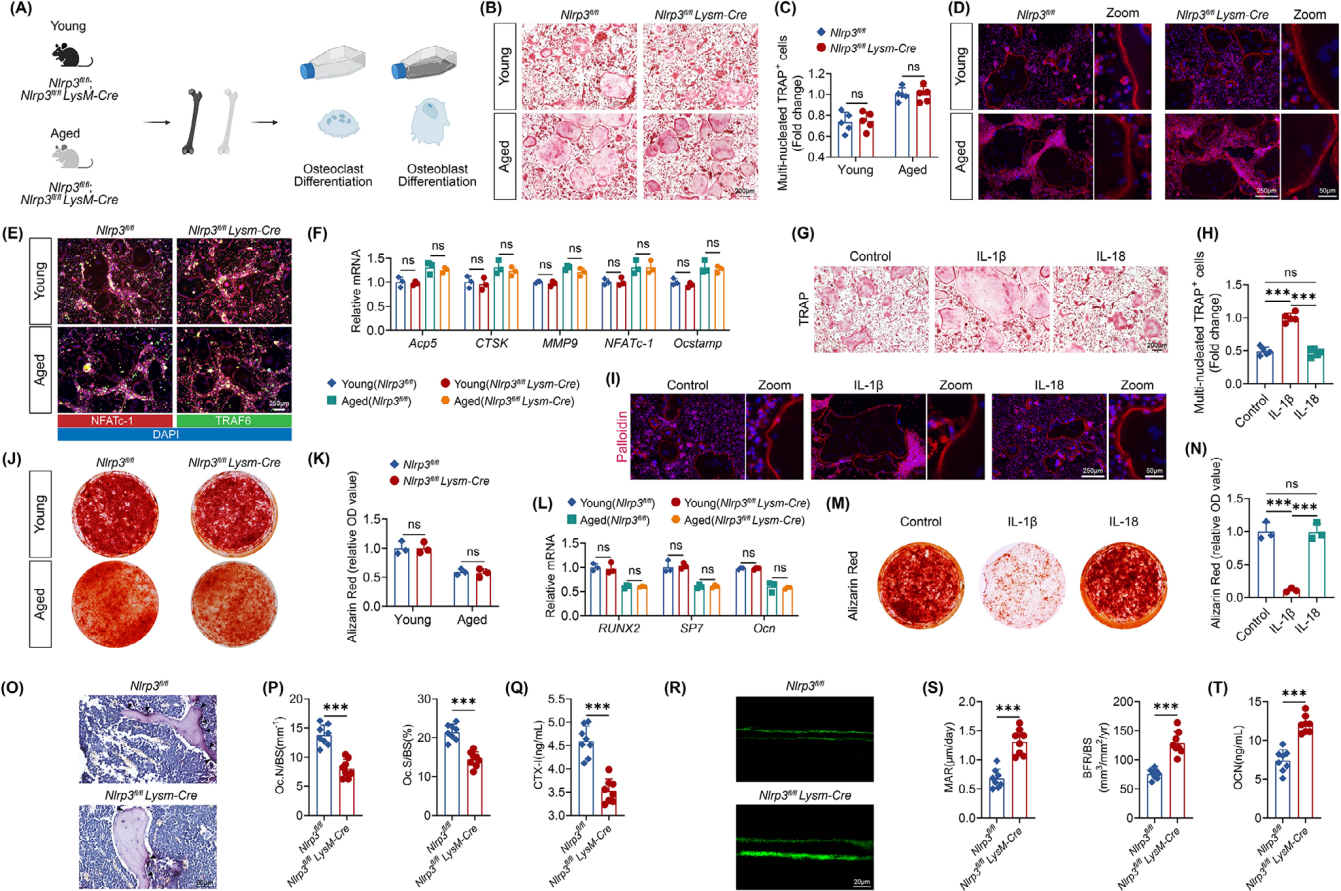
IL‐1β promoted osteoclastogenesis and inhibited osteogenesis (A) Schematic of osteoclast and osteoblast differentiation in *Nlrp3^fl/fl^LysM‐Cre* and their littermate control young and old mice. (B,C) Images of TRAP staining (B) and quantification (C) of osteoclasts differentiated from bone marrow cells of *Nlrp3^fl/fl^ LysM‐Cre* and their littermate control young and old mice (*n* = 5). (D) Fluorescent images of F‐actin staining of osteoclasts labeled by phalloidin. (E) Representative immunofluorescent images of NFATc‐1 and TRAF6 of osteoclasts. (F) qPCR analysis of genes related to osteoclast differentiation and bone resorption ability (*n* = 3). (G,H) Images of TRAP staining (H) and quantification (I) of osteoclasts treated with IL‐1β or IL‐18, respectively (*n* = 5). (I) Representative fluorescent images of phalloidin labeled F‐actin of osteoclasts treated with IL‐1β or IL‐18, respectively (*n* = 5). (J,K) Representative Alizarin Red staining images (J) and quantitative analysis (K) of BMSCs (*n* = 5). (L) qPCR analysis of osteoblast‐related marker genes (*n* = 3). (M,N) Representative Alizarin Red staining images (M) and quantitative analysis (N) of BMSCs (*n* = 5). (O) TRAP staining of femur sections. Black arrows indicate osteoclasts. (P) Quantitative analysis of osteoclasts surface per bone surface (Oc.S/BS) and osteoclasts number per bone surface (Oc.N/BS). *n* = 8. (Q) Serum level of CTX in *Nlrp3^fl/fl^* and *Nlrp3^fl/fl^LysM‐Cre* mice. *n* = 8. (R) Representative image of calcein double labeling of femur trabecular bones. Scale bar: 20 µm. (S) Quantitative analysis of mineral apposition rate and bone formation rate (*n* = 8). (T) Serum level of OCN in *Nlrp3^fl/fl^* and *Nlrp3^fl/fl^LysM‐Cre* mice (*n* = 8). Data were shown as means ± SD. Each point in scatter plots represented one individual. All experiments were conducted with three or more independent biological replicates per group. Significance was assessed by two‐tailed unpaired t‐test (C,K,O,P,Q,R,S,K), or One‐way ANOVA (Tukey) analysis (F,H,L,N): ns (no significance), ^***^ *p* < 0.001.

Considering that both macrophages and osteoclasts were derived from the mononuclear phagocyte system and the expression of NLRP3 had been implied to be associated with osteoclasts and osteoblasts differentiation [22, 23, 24], further investigations were needed to determine whether delayed skeletal aging induced by deletion of *Nlrp3* was depending on the activation of inflammasome or its direct effect on osteoclasts and osteoblasts differentiation. On this account, we first obtained mononuclear cells and mesenchymal stem cells from bone marrow to induce osteoclasts and osteoblasts differentiation (Figure 2A). However, *Nlrp3* conditional knockout in myeloid cells did not disturb the osteoclastogenesis. We observed a comparable number of TRAP‐positive, multinucleated cells (Figure 1B,C). To confirm the impact of *Nlrp3* deletion on the ability of bone resorption, we also perform the F‐actin staining to observe podosome arrangement. However, we still did not detect any differences in podosome belts or actin ring formation (Figure 2D). Consistently, osteoclast marker expression at both protein and mRNA levels followed this pattern. (Figure 2E,F). Next, we evaluated how *Nlrp3* deletion indirectly regulated bone homeostasis. Given that mature IL‐1β and IL‐18 were the main products of NLRP3 inflammasome activation, we then examined their potentiality for osteoclast differentiation. Unlike the direct effect of NLRP3 on osteoclasts differentiation, treatment with IL‐1β but not IL‐18 led to more TRAP^+^ multinucleated cell generation (Figure 2G,H). However, no obvious changes were observed in the podosome arrangement (Figure 2I).

To further unveil the underlying mechanism, we subsequently analyzed BMSCs (Bone marrow mesenchymal stem cells) osteogenic differentiation. It was demonstrated that *Nlrp3* ablation of myeloid cells did not affect osteogenesis as evidenced by a comparable expression of osteoblast markers and mineralization (Figure 2J–L). On the contrary, intervention with IL‐1β impaired osteogenic differentiation and mineralization of osteoblasts (Figure 2M,N). Besides, it was demonstrated that IL‐18 was still not involved in osteogenesis (Figure 2M,N). To exclude the influence of the *Nlrp3* deletion on osteoclastogenesis in vivo, we constructed an inflammatory model through LPS intervention in both wild‐type (*Nlrp3^fl/fl^*) and *Nlrp3* conditional knockout mice (*Nlrp3^fl/fl^LysM‐Cre*). Results indicated that NLRP3 deficiency significantly compromised osteoclast differentiation and function compared to wild‐type controls as evidenced by a lower level of osteoclasts number and serum of CTX‐I (Figure 2O–Q). More importantly, we also observed a higher bone formation rate and OCN in *Nlrp3^fl/fl^LysM‐Cre* mice (Figure 2R–T). Thus, our results ruled out the possibility that deletion of *Nlrp3* in myeloid cells had any direct effect on osteogenesis or osteoclastogenesis and confirmed that senescent macrophages were more susceptible to activating the NLRP3 inflammasome, secrete more cytokines of IL‐1β, and lead to skeletal aging.

### 2.2 Aging Led to Sirt3 Deficiency and Enhanced the Activity of Inflammatory Signaling Pathway

**FIGURE 3:**
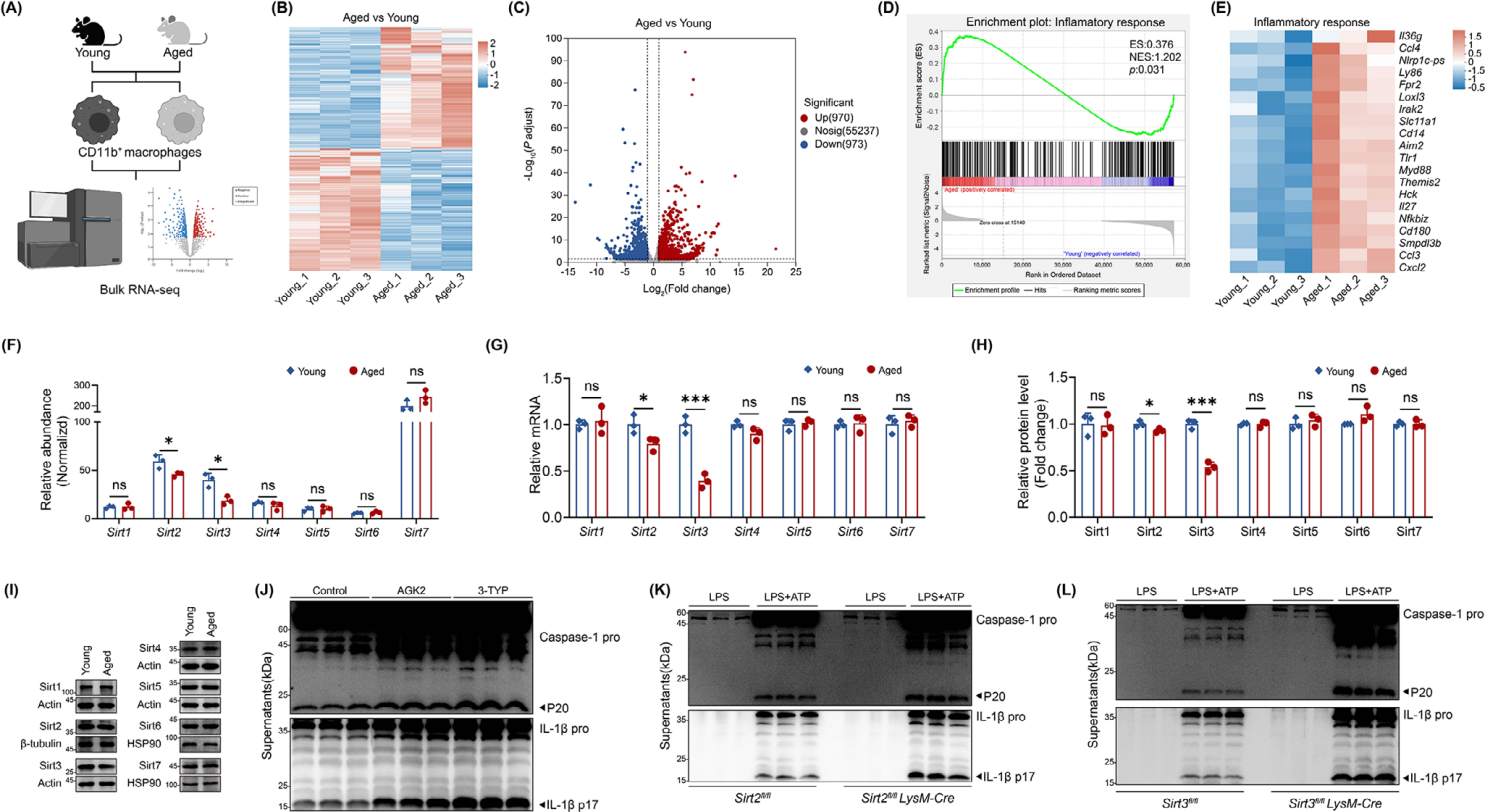
Aging led to the decrease of Sirt3 in macrophages and exacerbated the NLRP3 inflammasome activation. (A) Schematic diagram of bulk RNA seq of macrophages derived from the dataset of GSE98249. (B) Heatmap of genes that significantly differentially expressed in macrophages (aged macrophages vs. young macrophages). (C) The volcano plot of genes in young and senescent macrophages. (D) GSEA analysis of inflammatory response‐associated genes. (E) Heatmap of some inflammatory response‐associated genes. (F) Expression of Sirtuin family genes in macrophages of GSE98249. (G) qPCR analysis of Sirtuin family genes in macrophages (*n* = 3). (H,I) The protein level (O) and quantification (P) of the Sirtuin family in macrophages (*n* = 3). (J) The protein level of caspase‐1 and IL‐1β of BMDMs treated with none (Control), AGK2, or 3‐TYP. (K) BMDMs from *Sirt2^fl/fl^* or *Sirt2^fl/fl^* LysM‐Cre mice were primed with LPS (250 ng/mL) for 4h and then stimulated with ATP (3 mm) for 1h. Supernatants were collected for the immunoblot of caspase‐1 and IL‐1β. (L) BMDMs from *Sirt3^fl/fl^* or *Sirt3^fl/fl^* LysM‐Cre mice were primed with LPS (250 ng/mL) for 4h and then stimulated with ATP (3mM) for 1h. Supernatants were collected for the immunoblot of caspase‐1 and IL‐1β. Results are presented as mean ± SD. Each point in scatter plots represented one individual. All experiments were conducted with three or more independent biological replicates per group. Significance was assessed by two‐tailed unpaired t‐test: ns (no significance), ^*^ *p* < 0.05, ^**^ *p* < 0.01, and ^***^ *p* < 0.001.

After confirming that the immune dysfunction of macrophages during aging mediated age‐related osteoporosis, we next tried to investigate the underlying mechanism. First, we reanalyzed mRNA profiles of bone marrow CD11b^+^ macrophage of aged (24 months) and young (4 months) mice from a previous study (GSE98249) (Figure 3A) [25]. Significant differences of genes expression between macrophages from young and aged mice could be observed by the heatmap, volcano plot, and PCA analysis (Figure 3B,C; Figure S3A). We observed that genes associated with inflammatory response were significantly up‐regulated in aged macrophages, which was in accord with aggravated activation of NLRP3 inflammasome (Figure 3D,E; Figure S3B,C). Considering protective roles of longevity‐related genes in controlling inflammation, we analyzed the genes expression, including autophagy, HADCs, sirtuins, and so on [18, 26].

First, we found *Atg4b*, *Atg9b*, *Pik3c3*, and *Ulk2* were down‐regulated, while *Sqstm1* was highly expressed in aged macrophages compared with their young controls (Figure S3D,E). However, most autophagy‐related genes remained unchanged. Considering that autophagy had been implied to regulate NLRP3 inflammasome activation [27], we transfected young and aged BMDMs with mcherry‐GFP‐LC3 construct to monitor autophagic flux. GFP, the green fluorescent protein, was sensitive to acidic pH (quenched in lysosomes), while the red fluorescent protein was resistant to acidification (remained fluorescent in lysosomes). Thus, red puncta represented autolysosomes while yellow puncta indicated autophagosomes. We observed a comparable amount of autolysosomes both in young and aged macrophages (Figure S3F,G). Additionally, genes encoding AMPK and mTOR, the upstream signal pathway of autophagy, also showed no significant changes (Figure S3H). These results suggested that autophagy was not associated with aggravated NLRP3 inflammasome activation. Then, we focused on HDAC (Histone Deacetylase Inhibitor) family, which had been implied in the manipulation of immune response and aging‐related diseases. Among these HADCs, only *Hdac3* and *Hdac10* showed a decreased trend (Figure S3I). To verify whether deficiency of *Hadc3* or *Hdac10* mediated the high sensitivity of senescent macrophages, we treated BMDMs with their inhibitors, respectively. However, both RGFP966 (Hdac3 inhibitor) and Salvianolic acid B (Hdac10 inhibitor) even blocked IL‐1β release (Figure S3J), indicating that the decrease of *Hdac3* or *Hdac10* was not the reason of aggravated NLRP3 inflammasome activation. Consistent with an established study, butyrate and TSA, two HADC inhibitors, deactivated the NLRP3 inflammasome [28]. Apart from these, we also analyzed the expression level of genes that encoded FoxO family (*Foxo1*, *Foxo3*, *Foxo4*, *Foxo6*), oxidative defender Nrf2 (*Nfe2l2*), NAD^+^ consumer Parp1 (*Parp1*), telomerase reverse transcriptase (*Tert*), transcription factor Forkhead Box P1 (*Foxp1*), aging‐suppressor protein Klotho (*Kl*), and epigenetic regulator TET2 (*Tet2*). Among them, *Nfe2l2*, *Parp1*, and *Tet2* were significantly changed in macrophages derived from aged mice (Figure S3K,L). However, *Nfe2l2* and *Tet2* had been verified to be negatively correlated with NLRP3 inflammasome activation [4, 29]. Meanwhile, *Parp1* was proposed to promote NLRP3 inflammasome activation [30].

As the Sirtuins family was proposed to be a key regulator of aging as well as bone metabolism [31], we also examined the gene expression of *Sirt1* to *Sirt7* in young (4 months) and aged (24 months) macrophages (GSE98249). Interestingly, only *Sirt2* and *Sirt3* expression decreased with aging, while the other Sirtuins (*Sirt1*, *Sirt4*, *Sirt5*, *Sirt6*, and *Sirt7*) remained unchanged (Figure 3F). To further verify these findings, we examined the mRNA level of Sirtuins in macrophages. As speculated, all Sirtuins were unchanged except for *Sirt3* and *Sirt2* in macrophages from old mice, with a similar trend of dataset GSE98249 (Figure 3G). However, only Sirt3 but not Sirt2 was significantly decreased in the protein level of macrophages isolated from young (4 months) and old mice (24 months) (Figure 3H,I). More importantly, both Sirt2 inhibitor AGK2 and Sirt3 inhibitor 3‐TYP aggravated the NLRP3 inflammasome activation (Figure 3J; Figure S3M). Similar results were also observed in macrophages deficient in *Sirt2* or *Sirt3* (Figure 3K,L; Figure S3N,O). However, in contrast to the approximately 60% decrease in *Sirt3*, *Sirt2* was only reduced by about 20% in aged macrophages (Figure 3F,G). To verify the impact of varying degrees of decline in Sirt2 and Sirt3 on the NLRP3 inflammasome activation, *Sirt2* or *Sirt3* was knocked down by different concentrations of siRNA, respectively. Notably, about 25% knockdown of *Sirt2* showed no significant effect on NLRP3 inflammasome activation, whereas reducing *Sirt3* to approximately 25% of its normal level was sufficient to enhance the secretion of IL‐1β (Figure S3P–S). Moreover, when both Sirt3 and Sirt2 were reduced by about 50% respectively, IL‐1β release was significantly increased (Figure S3P–S). Collectively, these results suggested that a deficiency of Sirt3 might be the major factor that mediated the exacerbated activation of NLRP3 inflammasome.

### 2.3 Deficiency of Sirt3 Aggravated NLRP3 Inflammasome Activation and Accelerate Skeletal Aging

**FIGURE 4:**
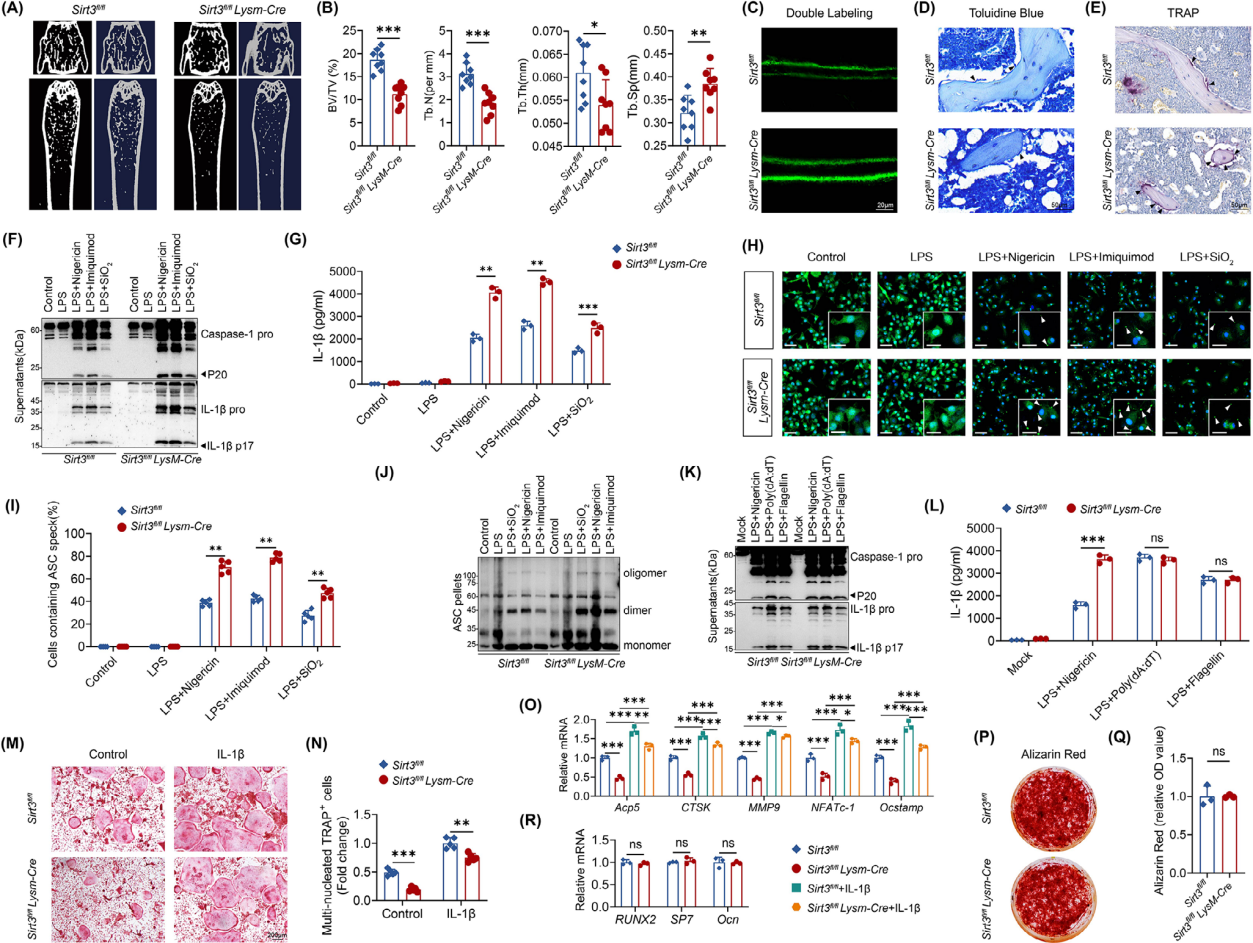
Lack of Sirt3 enhanced the NLRP3 inflammasome activation and accelerated bone loss. (A) 3D‐reconstructed MicroCT images of vertebra and femur. (B) Micro‐CT‐based 3D morphometric evaluation of femoral trabecular architecture quantified BV/TV, Tb.Sp, Tb.Th, and Tb.N (*n* = 8). (C) Fluorescence micrographs of calcein double‐labeled trabecular bone sections of femur. (D) TB staining of femur sectuons. Black arrows indicate osteoblasts. (E) TRAP staining of femur sectuons. Black arrows indicate osteoclasts. (F,G) Following LPS priming, BMDMs were exposed to distinct NLRP3 inflammasome activators: Nigericin, SiO_2_, or Imiquimod, respectively. Supernatants were subjected to the analysis of caspase‐1 and IL‐1β by western blot (F), and the secretion of IL‐1β by ELISA (G) (*n* = 3). (H,I) ASC speck formation was visualized by confocal immunofluorescence microscopy (H) and quantified as puncta per cell (I) in LPS‐primed BMDMs following stimulation with Nigericin, SiO_2_, or imiquimod (*n* = 5). (J) Following LPS priming, BMDMs were exposed to distinct NLRP3 inflammasome activators: Nigericin, SiO_2_, or Imiquimod, respectively. ASC oligomerization was analyzed by immunoblot. (K,L) Following LPS priming, BMDMs were exposed to distinct NLRP3 inflammasome activators: Nigericin, flagellin, or poly (dA:dT), respectively. Supernatants were subjected to the analysis of caspase‐1 and IL‐1β by western blot (K), and secretion of cytokine IL‐1β by ELISA (L) (*n* = 3). (M,N) Images of TRAP staining (M) and quantification (N) of osteoclasts differentiated from bone marrow cells of *Sirt3^fl/fl^LysM‐Cre* and their littermate control mice (*n* = 5). (O) qPCR analysis of genes related to osteoclast differentiation and bone resorption ability (*n* = 3). (P,Q) Representative images (P) and quantitative analysis (Q) of Alizarin Red staining of BMSCs (*n* = 5). (R) qPCR analysis of osteoblast‐related marker genes (*n* = 3). Results are presented as mean ± SD. Each point in scatter plots represented one individual. All experiments were conducted with three or more independent biological replicates per group. Significance was assessed by two‐tailed unpaired t‐test (B,G,I,L,N,R,Q) or One‐way ANOVA (Tukey): ns (no significance), ^*^ *p* < 0.05, ^**^ *p* < 0.01, and ^***^ *p* < 0.001.

To determine whether Sirt3 deficiency enhanced NLRP3 inflammasome activation and subsequently led to bone loss, we first crossed *Sirt3* floxed mice (*Sirt3^fl/fl^*) with *LysM‐Cre* mice to generate mice deletion of *Sirt3* in myeloid cells (*Sirt3^fl/fl^LysM‐Cre*) and analyzed the bone phenotype. We observed homozygous knockout of *Sirt3* in myeloid cells led to an accelerated bone loss with lower levels of trabecular bone volume, number, and thickness, but a higher level of trabecular bone separation (Figure 4A,B). Besides, histological section staining also revealed opposite results in mice with deletion of *Nlrp3* in myeloid cells. *Sirt3* knockout in myeloid cells led to more fat accumulation in bone marrow, more TRAP^+^ osteoclastic cells, and a high level of Serum CTX but fewer osteoblasts on the bone surface, decreased new bone formation, and lower bone formation rate (Figure 4C–E; Figure S4A–G).

To test whether *Sirt3* mediated the susceptibility of NLRP3 inflammasome during aging, we further obtained macrophages from WT (*Sirt3^fl/fl^*) and *Sirt3^fl/fl^ LysM‐cre* mice, and activate the NLRP3 inflammasome with nigericin, Imiquimod, or SiO_2_, respectively. We observed that deletion of *Sirt3* promoted the generation of cleaved caspase‐1 (P20) and IL‐1β (P17) upon the stimulation with structurally diverse NLRP3 agonists, but not the transfection with flagellin or Poly(dA:dT), indicating that Sirt3 deficiency specifically mediated aberrant activation of the NLPR3 inflammasome rather than the other kind of inflammasome (Figure 4F,G,K,L; Figure S4H–L). Additionally, deletion of *Sirt3* also promoted ASC speck formation and oligomerization (Figure 4H–J). As discussed above, *Sirt3* deficient macrophages exhibited an identical phenotype in NLRP3 inflammasome as macrophages isolated from aged mice.

Due to the fact that both osteoclasts and macrophages are of myeloid origin (Figure S4M), we performed osteoclast induction to confirm whether deletion of *Sirt3* affected osteoclastogenesis. However, knockout of *Sirt3* in myeloid cells disturbed the differentiation of osteoclasts as evidenced by a reduced number of TRAP^+^ cells and expression of marker genes (Figure 4M–O). Unlikely, osteoblasts differentiation remained unchanged. Both ARS staining and osteoblast‐related markers presented with a similar trend (Figure 4P–R). Based on the above experimental results, we found that myeloid cells‐specific *Sirt3* deletion led to two contradictory outcomes: (1) First, in macrophages, Sirt3 deficiency exacerbated NLRP3 inflammasome activation and increases IL‐1β secretion, thereby promoting osteoclast differentiation and aggravating bone resorption; (2) Second, when Sirt3 was absent specifically in osteoclast precursors or mature osteoclasts, their differentiation was impaired, leading to reduced bone‐resorbing capacity (Figure S4N). Although Sirt3 deficiency impaired osteoclast differentiation, the presence of IL‐1β stimulation could still significantly increase the formation of multinucleated osteoclasts (Figure 4M–O). These results demonstrated that the *Sirt3* deficiency‐induced increase in IL‐1β secretion might exert a far stronger promotive effect on osteoclast differentiation than its intrinsic inhibitory effect on osteoclastogenesis. To confirm that osteoclast hyperactivation driven by exacerbated IL‐1β secretion from Sirt3‐deficient macrophages plays a dominant role in vivo, we treated myeloid‐specific Sirt3 conditional knockout (*Sirt3^fl/fl^LysM‐Cre*) mice with an IL‐1β neutralizing antibody (Anti‐IL‐1β). The results showed that IL‐1β neutralization significantly alleviated bone loss in *Sirt3^fl/fl^LysM‐Cre* mice and reduced serum levels of CTX‐I (Figure S4O–Q). Collectively, these results indicated that the indirect effect of Sirt3 deletion in macrophages (IL‐1β) on osteoclasts outweighs the direct effect of Sirt3 deletion on osteoclasts themselves. Besides, these results also confirmed our speculation that loss of Sirt3 during aging mediated the susceptibility of NLRP3 inflammasome and age‐related osteoporosis.

### 2.4 *Nlrp3* Knockout Abolished Negative Effects of Sirt3 Deficieny on Bone Metabolism

**FIGURE 5:**
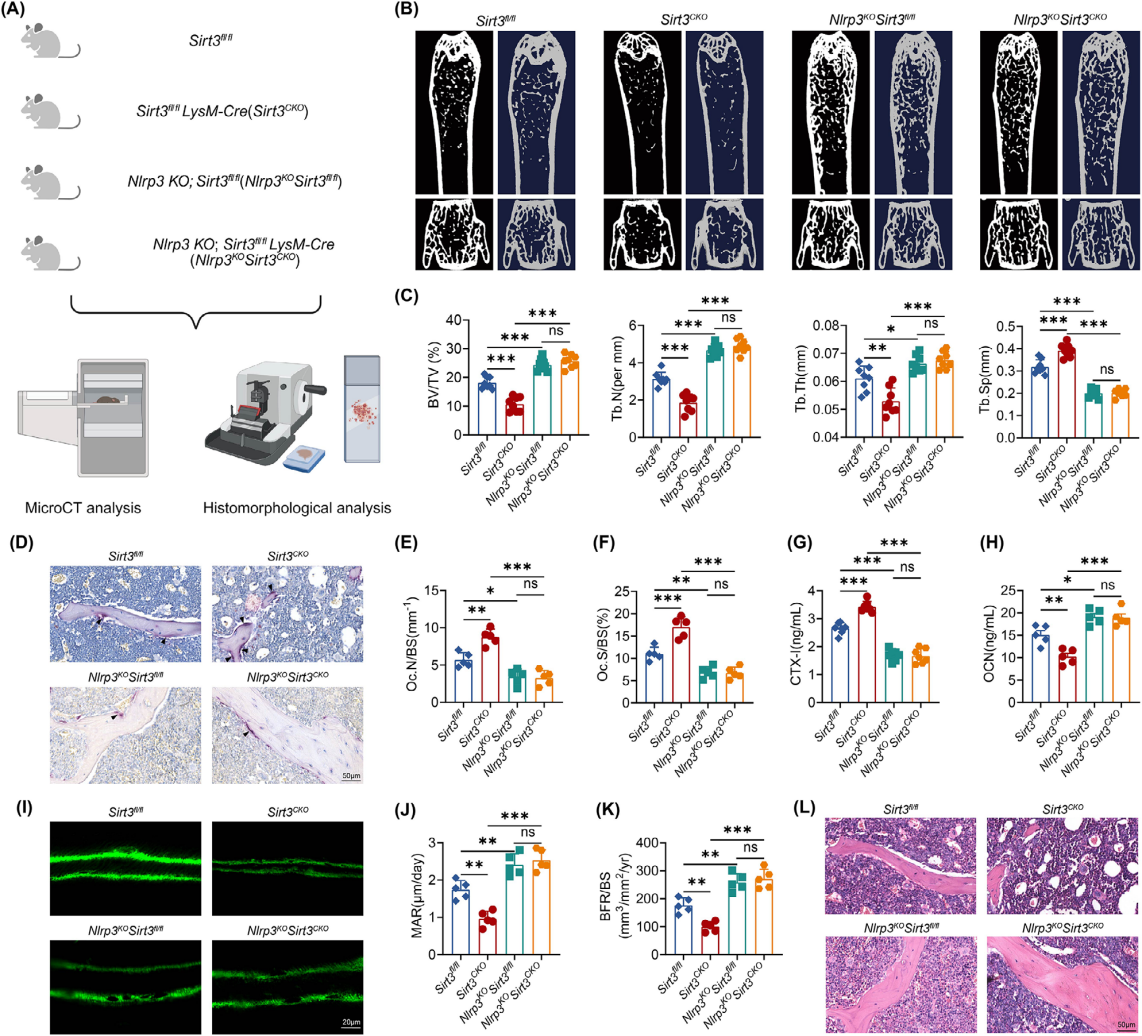
NLRP3 knockout abolished the negative effects of Sirt3 deficiency on bone mass. (A) Schematic diagram of double knockout mice experiments. (B) Representative vertebral and femoral images of micro‐CT. (C) Micro‐CT‐based 3D morphometric evaluation of femoral trabecular architecture quantified BV/TV, Tb.Sp, Tb.Th, and Tb.N (*n* = 8). (D–F) TRAP staining of femur sections (D), and histomorphometric assessment of osteoclasts number on trabecular surfaces of femur (N.Oc/BS) (*n* = 5) (E) and osteoclast coverage on trabecular surfaces of femur (Oc.S/BS) (*n* = 5). Black arrows indicate osteoclasts. (G,H) Serum level of CTX (G) and OCN (H) in *Sirt3^fl/fl^*, *Sirt3^CKO^*, *Nlrp3^KO^Sirt3^fl/fl^*, and *Nlrp3^KO^Sirt3^CKO^* mice (*n* = 5). (I–K) Fluorescence micrographs of calcein double‐labeled trabecular bone sections of femur (I) and quantification of mineral appositional rate (J) and bone formation rate (K) (*n* = 5). (L) HE staining of distal femur. Data were shown as means ± SD. Each point in scatter plots represented one individual. All experiments were conducted with three or more independent biological replicates per group. Significance was assessed by One‐way ANOVA (Tukey): ns (no significance), ^*^ *p* < 0.05, ^**^ *p* < 0.01, and ^***^ *p* < 0.001.

As discussed above, loss of Sirt3 during aging mediated the susceptibility of NLRP3 inflammasome activation of macrophages and age‐related osteoporosis. To further obtain in vivo evidence that age‐related osteoporosis induced by Sirt3 deficiency was a result of aggravated NLRP3 inflammasome activation, we crossed *Sirt3^fl/fl^ LysM‐cre* mice (*Sirt3^CKO^*) with *Nlrp3*‐null (*Nlrp3^KO^*) mice to generate mice with double knockout of *Nlrp3* and *Sirt3* (*Nlrp3^KO^Sirt3^CKO^*) (Figure 5A). *Nlrp3^KO^Sirt3^CKO^* mice reached adulthood with comparable body size and weight to controls at 16 months (Figure S5A). Different from *Sirt3^fl/fl^ LysM‐cre* mice, mice with double knock out of *Sirt3* and *Nlrp3* (*Nlrp3^KO^Sir3^CKO^*) revealed a healthy bone mass, as *Nlrp3 KO* (*Nlrp3^KO^Sirt3^fl/fl^*) controls and even better than *Sirt3^fl/fl^* mice (Figure 5B,C). Consistent with the micro‐CT analysis, we observed fewer TRAP‐positive osteoclasts on the trabecular bone surface and lower serum concentration of CTX in *Nlrp3^KO^Sirt3^CKO^* and *Nlrp3^KO^Sirt3^fl/fl^* mice compared with *Sirt3^fl/fl^* or *Sirt3^CKO^* mice (Figure 5D–G). In contrast to the inactivity of osteoclastogenesis, a marked increase in new bone formation rate and serum level of OCN were observed in *Nlrp3^KO^Sirt3^fl/fl^* and *Nlrp3^KO^Sirt3^CKO^* mice (Figure 5H–K). Besides, HE staining also revealed that both area and number of adipocytes were lower in *Nlrp3^KO^Sirt3^fl/fl^* and *Nlrp3^KO^Sirt3^CKO^* mice compared with *Sirt3^fl/fl^* or *Sirt3^CKO^* mice (Figure 5L). Besides, LPS‐induced sepsis, an acute inflammation model, was used to monitor macrophage‐mediated inflammatory response. TNF‐α production remained unchanged, whereas IL‐1β and IL‐18 release were abolished in both *Nlrp3^KO^Sirt3^fl/fl^* and *Nlrp3^KO^Sirt3^CKO^* mice (Figure S5B–D). More importantly, *Sirt3* ablation in myeloid cells increased serum level of IL‐1β and IL‐18, and this negative impact was almost interrupted by the deletion of *Nlrp3* (Figure S5B–D), indicating that Sirt3 was the up‐stream of NLRP3 inflammasome activation. Together, these results confirmed that Sirt3 loss during aging exacerbated NLRP3 inflammasome activation, which in turn mediated age‐related osteoporosis.

### 2.5 Sirt3 Promotes NLPR3 Degradation and Disrupts the Assembly of Inflammasome

**FIGURE 6:**
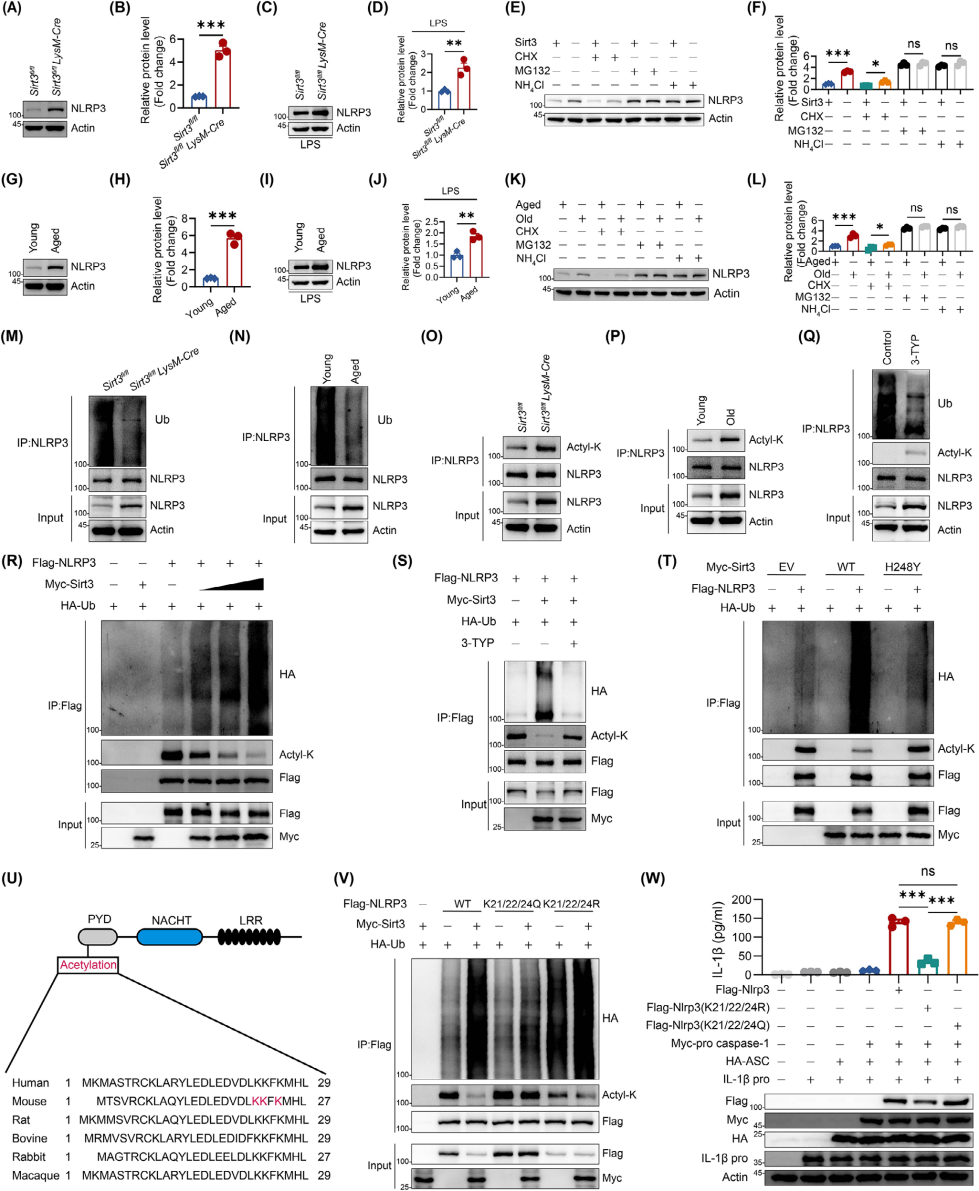
Sirt3 deficiency inhibited NLRP3 degradation and promoted NLRP3 inflammasome activation. (A‐B) The protein level (A) and quantitative analysis (B) of NLRP3 in BMDMs isolated from *Sirt3^fl/fl^* and *Sirt3^fl/fl^LysM‐Cre* mice. (C,D) BMDMs from *Sirt3^fl/fl^* and *Sirt3^fl/fl^LysM‐Cre* mice were stimulated with LPS for 4h. The expression (C) and quantitative analysis (D) of NLRP3 were analyzed. (E,F) Macrophages from *Sirt3^fl/fl^* and *Sirt3^fl/fl^ LysM‐Cre* mice were treated as indicated. The expression (E) and quantitative analysis (F) of NLRP3 were analyzed. (G,H) The protein level (G) and quantitative analysis (H) of NLRP3 in macrophages. (I,J) BMDMs were stimulated with LPS for 4h. The expression (I) and quantitative analysis (J) of NLRP3 were analyzed. (K,L) Macrophages of were treated as indicated. The expression (K) and quantitative analysis (L) of NLRP3 were analyzed. (M,N) NLRP3 ubiquitination in macrophages derived from *Sirt3^fl/fl^* and *Sirt3^fl/fl^ LysM‐Cre* mice (M) or young and aged mice (N) was assessed by immunoblotting. (O,P) NLRP3 ubiquitination and acetylation in macrophages derived from *Sirt3^fl/fl^* and *Sirt3^fl/fl^ LysM‐Cre* mice (O) or young and aged mice (P) were assessed by immunoblotting. (Q) NLRP3 ubiquitination and acetylation in macrophages treated as indicated were assessed by immunoblotting. (R) HEK293T cells co‐transfected with Flag‐NLRP3 and HA‐ubiquitin were exposed to escalating Myc‐Sirt3 concentrations. Anti‐Flag immunoprecipitates subsequently underwent immunoblotting to detect Ubiquitination and acetylation states. (S) Flag‐NLRP3 ubiquitination and acetylation were assessed in Myc‐Sirt3 and HA‐ubiquitin expressing HEK293T cells treated with or without 3‐TYP inhibitor. (T) HA‐ubiquitin expressing HEK293T cells were transfected with Flag‐NLRP3, and Myc‐Sirt3 or Myc‐Sirt3 H248Y as indicated. The immunoprecipitates of Flag‐NLRP3 were collected for the analysis of ubiquitination and acetylation by immunoblot. (U) The alignment of NLRP3 orthologs and the scheme of NLRP3 domain structure. The acetylated lysine (K) residue was marked in red. (V) HEK293T cells were transfected with Myc‐Sirt3, HA‐ubiquitin, Flag‐NLRP3, Flag‐NLRP3 K21/22/24Q, and Flag‐NLRP3 K21/22/24R as indicated.The Flag‐NLRP3 immunoprecipitates were collected for the analysis of ubiquitination and acetylation by immunoblot. (W) HEK293T cells were reconstituted by transfection with Flag‐NLRP3, Myc‐pro‐caspase‐1, HA‐ASC, pro‐IL‐1b, Flag‐NLRP3 (K21/22/24R), Flag‐NLRP3 (K21/22/24Q) as indicated, and then stimulated with nigericin. Supernatants were subjected to the analysis of the secretion of IL‐1β by ELISA (*n* = 3). Results are presented as mean ± SD. Each point in scatter plots represented one individual. All experiments were conducted with three or more independent biological replicates per group. Significance was assessed by two‐tailed unpaired *t*‐test (B,D,H,J), or One‐way ANOVA (Tukey) analysis (F,L,W): ns (no significance), ^*^ *p* < 0.05, ^**^ *p* < 0.01, and ^***^ *p* < 0.001.

Given that deletion of Sirt3 specifically aggravated NLRP3 inflammasome activation rather than other kinds of inflammasome (Figure 4K), we first examined the mRNA and protein levels of NLRP3. To our surprise, no significant changes could be observed in transcriptional level, while the protein level of NLRP3 was overexpressed in Sirt3 KO macrophages compared with Sirt3 sufficient macrophages (Figure 6A,B; Figure S6A). More importantly, the same results can also be observed upon the intervention with LPS (Figure 6C,D). Thus, we speculated that differences in the degradation of protein might be the result of higher levels of NLRP3 in Sirt3 KO macrophages. To verify our hypothesis, we used protein synthesis pan inhibitor CHX (Cycloheximide), proteasome pathway degradation inhibitor MG132 (Z‐Leu‐Leu‐Leu‐al), or lysosome pathway degradation inhibitor NH_4_Cl (Ammonium Chloride), respectively. As we speculated, there was a comparable amount of NLRP3 in Sirt3 sufficient and KO macrophages when the protein degradation pathway was blocked by MG132 or NH_4_Cl. However, NLRP3 was degraded much more quickly in Sirt3 sufficient macrophages in the presence of CHX (Figure 6E,F). These results suggested Sirt3 promoted the degradation of NLRP3. Then, we also detected mRNA and protein expression of NLRP3 in BMDMs isolated from young and old mice. Consistent with the results of Sirt3 sufficient and deficient macrophages, the mRNA level of *Nlrp3* slightly elevated without statistical significance in aged BMDMs compared with young BMDMs, while the protein level of NLRP3 of aged BMDMs was increased both in untreated and LPS stimulation (Figure 6G–J; Figure S6B,C). More importantly, it was also revealed that protein degradation, but not synthesis mediated the higher expression of NLRP3 in aged BMDMs (Figure 6K,L).

Next, we put our emphasis on the slow degradation rate of NLRP3 mediated by Sirt3 deficiency or aging. Due to the fact that both proteasome and lysosome pathways require ubiquitin labeling before degradation, we thus analyzed the ubiquitination level of NLRP3 in aged or young and Sirt3 sufficient or deficient BMDMs. As we speculated, aging and Sirt3 deficiency greatly decreased the ubiquitination level of NLRP3 (Figure 6M,N). Given that Sirt3 is a multifunctional deacetylase, which regulates protein expression by directing post‐translational modification. To verify whether Sirt3 degraded NLRP3 by its potential as a deacetylase, we further detected the acetylation level of NLRP3. Similar to Sirt3 KO macrophages, BMDMs from Old mice exhibit a higher acetylation level of NLRP3 (Figure 6O,P). Of note, we also found that Sirt3 inhibitor 3‐TYP could prevent NLRP3 degradation and ubiquitination in BMDMs, while promoting NLRP3 acetylation (Figure 6Q). Collectively, with the result that Sirt3 KO also led the resistance of NLPR3 to degradation via ubiquitin, we speculated that Sirt3 deficiency may abolish the ability of macrophages to deacetylate NLRP3, which in turn losing control of the ubiquitin‐dependent degradation of NLRP3.

To validate the dual regulatory role of Sirt3 in NLRP3 post‐translational modifications, HEK293T cells were co‐transfected with Flag‐NLRP3, Myc‐Sirt3, and HA‐ubiquitin. Immunoblot analysis revealed that Sirt3 dose‐dependently enhanced NLRP3 ubiquitination while reducing its acetylation, which could be abolished by the Sirt3 inhibitor 3‐TYP (Figure 6R,S). To reconfirm the Sirt3‐mediated deacetylation regulated NLRP3 expression, we also transfected HEK293T cells with Myc‐Sirt3, Flag‐NLRP3, or myc‐Sirt3 mutant (H284Y) plasmids. The results showed that Sirt3 WT plasmid, but not Sirt3 mutant plasmids without deacetylation function limited the expression of NLRP3 (Figure 6T). Besides, the ubiquitination level of NLRP3 was also blocked when transfected with Sirt3 mutant (H284Y) plasmid (Figure 6T). Considering that previous studies reported that K21/22/24 were the sites of NLRP3 acetylation (Figure 6U), we then constructed NLRP3 mutants in which lysine (K) 21/22/24 residues were replaced by glutamine (Q) to mimic acetylation or arginine (R) to mimic deacetylation [17, 32]. Our results revealed that K21/22/24R mutant promoted NLRP3 degradation as Sirt3 WT plasmid, while K21/22/24Q was resistant to degradation (Figure 6V). Meanwhile, K21/22/24R mutant increased the ubiquitination level of NLRP3. In contrast, K21/22/24Q mutation led to the reduction of NLRP3 ubiquitination (Figure 6V). Taken together, these results further confirmed that loss of Sirt3 during aging prevented NLRP3 degradation.

Then we transfected HEK293T cells with essential components of NLRP3 inflammasome, or NLRP3 mutants to explore the influence of Sirt3‐mediated deacetylation and ubiquitination in the assembly of inflammasome complex. With 48h reconstitution of the NLRP3 inflammasome, HEK293T cell could successfully release IL‐1β. Different with NLRP3 WT, K21/22/24R mutant nearly abolished the secretion of IL‐1β, while K21/22/24Q mutant remained unchanged of IL‐1β production (Figure 6W).

### 2.6 Construction of Engineered Apoptotic Bodies for Delivering Sirt3

**FIGURE 7:**
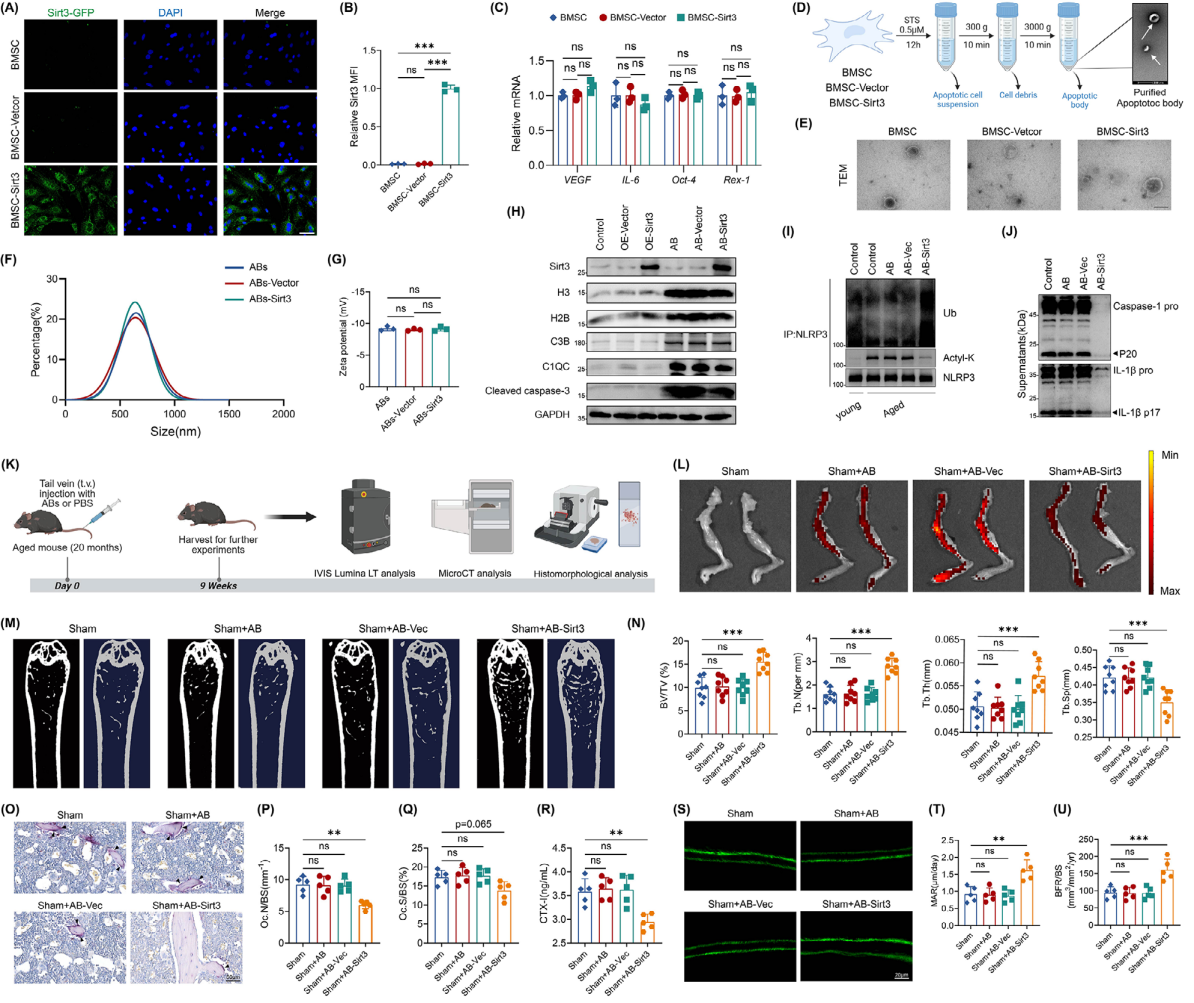
Sirt3 genetically engineered apoptotic bodies delayed age‐related bone loss. (A) Representative fluorescent images of BMSCs transfected with lentivirus encoding the Sirt3 gene tagged by GFP. (B) Quantitative analysis of the mean fluorescence intensity (MFI) of Sirt3 (*n* = 3). (C) qRCR analysis of *VEGF*, *IL‐6*, *Oct‐4* and *Rex‐1* in BMSCs, BMSC‐Vector, and BMSC‐Sirt3. (D) The schematic diagram for the isolation of ABs from BMSC, BMSC‐Vector, and BMSC‐Sirt3. Black arrows indicate osteoblasts. (E) Representative TEM (transmission electron microscope) images of ABs, ABs‐Vector, and ABs‐Sirt3. (F) The size distribution of ABs, ABs‐Vector, and ABs‐Sirt3. (G) The zeta potentials of the surface of ABs, ABs‐Vector, and ABs‐Sirt3 (*n* = 3). (H) Immunoblot analysis of H3, H2B, C3B, C1qc, cleaved caspase‐3. (I) BMDMs were primed with LPS in the presence of ABs, ABs‐Vector, or ABs‐Sirt3, respectively, and then stimulated with ATP. The NLRP3 immunoprecipitates were collected for the analysis of ubiquitination and acetylation. (J) BMDMs were primed with LPS in the presence of ABs, ABs‐Vector, or ABs‐Sirt3. Supernatants were subjected to the analysis of caspase‐1 and IL‐1β by Western blot. (K) The schematic diagram for the treatment of osteoporosis with ABs, ABs‐Vector, or ABs‐Sirt3, respectively. (L) ABs, ABs‐Vector, and ABs‐Sirt3 were labeled with the near‐infrared fluorophore and intravenously injected for 6h. The Ex vivo fluorescence images were captured by IVIS Lumina LT. (M) Representative femoral images of micro‐CT. (N) Quantitative analysis of femoral trabecular parameters: BV/TV, Tb.Sp, Tb.Th, Tb.N (*n* = 8). (O–Q) TRAP staining of femur sections (O) and histomorphometric assessment of osteoclast number on trabecular surfaces of femur (N.Oc/BS) (P) and osteoclast coverage on trabecular surfaces of femur (Oc.S/BS) (Q). (R) Serum level of CTX in Sham, Sham+ABs, Sham+ABs‐Vector, and ABs‐Sirt3 mice (*n* = 5). (S–U) Fluorescence micrographs of calcein double‐labeled trabecular bone sections of femur (S) and quantification of mineral appositional rate (T) and bone formation rate (U). Data were shown as means±SD. Each point in scatter plots represented one individual. All experiments were conducted with three or more independent biological replicates per group. Significance was assessed by One‐way ANOVA (Tukey): ns (no significance), ^**^ *p* < 0.01, and ^***^ *p* < 0.001, by One‐way ANOVA (Tukey's post hoc) analysis.

Given that Sirt3 lacked of specific activators and gene therapy still faced great challenges, we attempted to construct engineered extracellular vesicles to deliver Sirt3. Numerous studies showed that extracellular vesicles, one of the most promising therapeutic platforms, could be genetically engineered to express specific proteins and regulate cellular homeostasis [33]. As a special component of extracellular vesicles, apoptotic bodies also possess the same functions. Apart from these properties, apoptotic bodies, like apoptotic cells, are rich in phosphatidylserine (PS) and can be recognized and phagocytized by macrophages, which means apoptotic bodies can efficiently target macrophages without any modifications [21]. Thus, we first obtained BMSCs and confirmed by the flow cytometry analysis (Figure S7). Then, we infected BMSCs with lentivirus encoding Sirt3 and obtained high purity of Sirt3‐expressing BMSCs by puromycin selection (Figure 7A,B). The morphology, activity factors, and the expression of stem cell differentiation‐related genes were not altered in Sirt3‐expressing BMSCs (Figure 7C; Figure S8A). Then, we treated Sirt3‐expressing BMSCs with staurosporine to induce apoptosis and purified apoptotic bodies according to previous studies (Figure 7D). Transmission electron microscope (TEM) showed a round‐shaped morphology of apoptotic bodies with a diameter ranging from 105 to 1106 nm (Figure 7E,F). The zeta potential of apoptotic bodies was −9.21 mV (Figure 7G). Apoptotic bodies were identified by specific markers such as histone 2B (H2B), histone 3 (H3), C3B, C1QC, and cleaved caspase‐3 (Figure 7H). Our results showed that the Sirt3 protein concentration in apoptotic bodies derived from Sirt3‐overexpressing bone marrow mesenchymal stem cells (ABs‐Sirt3) was 352.2 ± 15.085 pg in 100 µg of ABs‐Sirt3 (Figure S8B). Compared to apoptotic bodies derived from non‐overexpressing cells, the loading efficiency increased by approximately 62‐fold (Figure S8B). Furthermore, flow cytometric analysis revealed that apoptotic bodies were almost completely phagocytosed by macrophages within 4 h (Figure S8C). More importantly, Sirt3 was highly expressed in apoptotic bodies transfected MSCs compared to those without transfection (Figure 7H; Figure S9A). These data indicated that Sirt3 genetically engineered apoptotic bodies were successfully constructed.

Next, we investigated whether Sirt3 genetically engineered apoptotic bodies were capable of inhibiting NLRP3 inflammasome activation. Macrophages were treated with ABs, ABs‐Vector (ABs‐Vec), or ABs‐Sirt3 and then activated NLRP3 inflammasome via LPS+ATP. As Figure 7I shows, treatment with ABs‐Sirt3 also led to the ubiquitination and deacetylation of NLRP3 (Figure 7I). Notably, intervention with ABs‐Sirt3 but not ABs or ABs‐Vec limited the cleavage of caspase‐1 and IL‐1β (Figure 7J). These results demonstrated ABs‐Sirt3 could suppress NLRP3 inflammasome activation.

Then, we attempted to verify its therapeutic potentiality on age‐related osteoporosis. The experimental schedule is shown in Figure 7K. To determine the distribution of Sirt3 genetically engineered apoptotic bodies in vivo, mice were intravenously injected with fluorescence‐labeled apoptotic bodies. We observed that these fluorescence‐labeled apoptotic bodies were possessed with a good natural tropism for bone (Figure 7L). The bone phenotype of aged mice treated with different ABs was analyzed by multiple methods. From the images of Micro‐CT 3D construction, we observed a delayed bone loss induced by aging in old mice treated with ABs‐Sirt3 compared with mice treated with ABs or ABs‐Vec, as there were more trabecular bone numbers, trabecular bone volume, but a lower level of trabecular bone separation (Figure 7M,N). Furthermore, TRAP‐positive cells were evidently decreased in aged mice treated with ABs‐Sirt3 (Figure 7O–Q). Correspondingly, the bone resorption marker of CTX of ABs‐Sirt3 treated mice was also at a lower level in contrast to ABs or ABs‐Vec treated mice (Figure 7R). Unlike the negative effects on the osteoclasts, ABs‐Sirt3 promoted the bone formation rate and new bone formation (Figure 7S–U). Collectively, these results suggested that Sirt3 genetically engineered apoptotic bodies were a qualified candidate for the treatment of age‐related osteoporosis.

## 3 Discussion

Currently, the global aging population is expanding at an unprecedented rate, creating profound social and economic challenges. By 2024, individuals aged 65 and older make up about 10.3% of the world's population, a notable increase compared to 5.5% in 1974. As of 2074, this percentage is expected to double to 20.7%, with the number of individuals aged 80 and above tripling during this period. With the accelerated pace of population aging, the incidence rate of age‐associated diseases, such as osteoporosis, cardiovascular diseases, neurodegenerative diseases, and so on, has been increasing markedly [34, 35, 36, 37]. Among them, age‐related osteoporosis is undoubtedly the most unique for its extremely high incidence rate. A systematic review and meta‐analysis of 40 studies involving 79 127 participants aged 50 to 85 reported an overall global prevalence of osteoporosis among elderly individuals at 21.7% (95% CI: 18.8%–25%) [38]. Current treatment measures for age‐related osteoporosis primarily aim is to improve bone boss and reduce fracture risk through the inhibition of osteoclasts or/and promotion of osteoblasts with pharmacological interventions. However, these measures cannot change unhealthy bone turnover or cellular aging, and may also be accompanied by some side effects [1, 39, 40].

More recently, the dysfunction of the immune system during aging, or inflammaging, has been regarded as a key risk factor in age‐associated diseases [41, 42]. With the advances of related research, blocking inflammatory pathways has been verified to alleviate age‐associated diseases. However, the impact of inflammaging on the skeletal system still remains many unknown mechanisms to be elucidated. Youm first reported that the 24‐month‐old Nlrp3^−/−^ mice presented with a higher bone mass and BMD compared with WT control mice, while this trend could not be observed at their young age [3]. More importantly, this potentiality in the regulation of bone metabolism only relied on canonical NLRP3 inflammasome activation rather than caspase‐11‐dependent noncanonical inflammasome. Given that Nlrp3 was expressed in various kinds of cells, the phenotype of *Nlrp3* ^−/−^ mice could not exactly reflect the influence of inflammaging on bone homeostasis. In this study, we crossed *Nlrp3* floxed mice with *LysM‐cre* mice to delete *Nlrp3* in macrophages. Consistent with Youm's study, the difference in bone phenotype was more pronounced with aging. Conditional deletion of *Nlrp3* in myeloid cells protected old mice from age‐related osteoporosis, whereas no discrepancy could be observed in young mice. Mechanistically, aging disturbed the degradation of NLRP3 and facilitated NLRP3 inflammasome activation. Except for our study, the NLRP3 inflammasome activation had also been implied to mediate the aging of other tissues and organs. For instance, Navarro‐Pando et al. revealed that aggravation of NLRP3 inflammasome activation was observed in the ovary during reproductive aging, while MCC950, a specific inhibitor of NLRP3, prevented ovarian aging [43]. Meanwhile, Zhang et al. reported that the activation of NLRP3 inflammasome promoted age‐related atrial fibrillation [44]. Despite the underlying activation mechanisms were different, the NLRP3 inflammasome has been universally recognized as pivotal in senescence‐associated disorders.

After confirming dysfunctional immune response of macrophages during aging contributed to age‐related osteoporosis, we next attempted to figure out the underlying mechanism that facilitated the NLRP3 inflammasome activation in senescent macrophages. Significant differences in gene expression between macrophages from young and old mice were observed by reanalyzing mRNA profiles from a previous study (GSE98249). Since the Sirtuins family was regarded as a key regulator of aging and bone metabolism, we examined the mRNA expression of each member. Interestingly, Sirt3 was downregulated in senescent macrophages compared with their young controls, whereas the other sirtuins remained unchanged. This was not a singular phenomenon that the expression of sirtuin members decreased in the cells of aging individuals. For example, the protein and mRNA levels of Sirt6 were dropped in old pre‐osteoclasts, while Sirt2 was downregulated in vascular cells [14, 45]. To verify whether Sirt3 mediated susceptibility of NLRP3 inflammasome activation and age‐related bone loss, we crossed *Sirt3^fl/fl^* mice with *LysM‐Cre* mice to generate *Sirt3^fl/fl^LysM‐Cre* mice. To our surprise, BMDMs from *Sirt3^fl/fl^ LysM‐Cre* mice also exhibited an aggravation of NLPR3 inflammasome activation compared with their WT controls. Similarly, the degradation of NLRP3 was also delayed in Sirt3 deficient macrophages. Corresponding with the in vitro studies, the bone phenotype of *Sirt3^fl/fl^LysM‐Cre* mice was analogous to old mice. More importantly, the double knockout of *Sirt3* and *Nlrp3* nearly abolished the negative effects of Sirt3 deficiency on bone metabolism. These results all validated our hypothesis that the expression of Sirt3 gradually decreased during aging, which in turn lost control of NLRP3 degradation and inflammasome activation.

Sex differences play a significant role in age‐related bone loss. In general, women experience more rapid and pronounced bone loss compared to men, particularly during the early postmenopausal period. This accelerated decline is primarily attributed to the sharp drop in estrogen levels following menopause, as estrogen plays a crucial protective role in maintaining bone homeostasis by suppressing osteoclast‐mediated bone resorption. Consequently, postmenopausal women are at a substantially higher risk of developing osteoporosis and suffering fragility fractures. In contrast, men undergo a more gradual decline in bone mass with aging, largely due to a slower, age‐related decrease in testosterone and bioavailable estrogen. However, our study only investigated the impact of exacerbated NLRP3 inflammasome activation in aged macrophages on male mice, and its effects in aged female mice remain unclear. To better translate these findings into clinical applications, future studies should thoroughly explore this mechanism in aged female mice.

EVs (Extracellular vesicles) such as microvesicles, exosomes, and apoptotic bodies have been regarded as promising candidates for delivery drugs [46, 47]. More recently, Xu et al. reported that engineered extracellular vesicles with high expression of SHP2 promoted mitophagy and alleviated Alzheimer's disease [33]. Considering that Sirt3 lacked specific agonists and the instability of gene therapy, we attempted to deliver the target protein through this biomedical platform. Compared with the other EVs, apoptotic bodies were specific to macrophages for the existence of phosphatidylserine, which could be recognized by macrophages and initiate an “eat‐me” signal. Based on these findings, we constructed engineered apoptotic bodies to deliver Sirt3 to macrophages. As we speculated, treatment with Sirt3‐enriched apoptotic bodies did not only suppress the NLRP3 inflammasome activation but also alleviated age‐related osteoporosis. These results indicated that the apoptotic bodies‐engineering technology provides a platform that could target macrophages to deliver specific proteins.

## 4 Experimental Section

### 4.1 Mice

Young, old, and aged C57BL/6J mice, *Nlrp3‐KO* mice (Strain S‐KO‐05210), *Nlrp3‐*floxed mice (Strain NO.S‐CKO‐06042), and *Sirt3*‐floxed mice (Strain S‐CKO‐12758) were purchased from Cyagen (Suzhou, China). All mice (C57BL/6J background) were bred and maintained under specific‐pathogen‐free (SPF) conditions at Shanghai Tenth's People's Hospital, with a 12:12 light‐dark cycle at 22°C–23°C, following Institutional Animal Care and Use Committee approved protocols. In the present study, only male mice and their littermate controls were used for experiments.

### 4.2 Bone Marrow Derived Macrophages (BMDMs) Preparation and Stimulation

BMDMs were isolated as previously described. Briefly, bone marrow cells were flushed from bilateral tibiae and femurs with α‐MEM medium (PM150421, Procell). Then, red blood cells were lysed and washed twice with PBS. Remnant cells were resuspended with α‐MEM medium containing 10% fetal bovine serum (f101, Vazyme) in the culture dish. The next day, non‐adherent cells were collected and plated into six‐well plate with a α‐MEM medium containing 10% fetal bovine serum and 40 ng/mL M‐CSF (CB34, Novoprotein). The culture medium was replaced every 3 days. After 5 to 7 days of culture, differentiated macrophages were subjected to further experiments. For the NLRP3 inflammasome activation, differentiated macrophages were primed with LPS (400ng/mL; SMB00610; Sigma) for 4h, and then stimulated with Nigericin (M7029; AbMole; USA), SiO2, or Imiquimod as indicated. The supernatants and cell lysates were collected for Western blot and ELISA analysis. For the activation of NLRC4 or AIM2 inflammasome, LPS‐primed BMDMs were stimulated with flagellin (1µg/mL; 6h; tlrl‐pafla; InvivoGen) or transfected dsDNA (1µg; 6h; tlrl‐patc; InvivoGen) as indicated. The supernatants were collected for Western blot analysis.

### 4.3 Peripheral Blood Mononuclear Cell (PBMCs) Isolation

PBMCs were isolated from 5 mL peripheral venous blood of three young and old healthy donors by using ficoll paque solution (Cytiva, Marlborough, MA). The study protocol received ethical approval from Ruijin Hospital, Shanghai Jiao Tong University School of Medicine (Approval No. 2024406), and all participants provided written informed consent prior to sample collection. The investigation was conducted in compliance with the ethical principles outlined in the Declaration of Helsinki. The demographics and clinical data are listed in Tables S2 and S3.

### 4.4 Western Blot

Cell lysates were prepared using RIPA buffer (Beyotime, Shanghai, China) supplemented with protease and phosphatase inhibitors. Protein concentrations were determined using a BCA assay kit (Beyotime). Following SDS‐PAGE separation (Beyotime), proteins were transferred to PVDF membranes (Beyotime). After blocking with Quick Block buffer (Beyotime) for 30 min, membranes were incubated with primary antibodies aginst NLRP3 (1:1000; AG‐20B‐0014; Adipogen life science), caspase‐1 (1:1000; AG‐20B‐0042; Adipogen life science), ASC (1:1000; 67824S; Cell Signaling Technology), IL‐1β (1:1000; GTX74034; Genetex), cleaved caspase‐3 (1:1000; 9661S; Cell Signaling Technology), Sirt1 (1:8000; RMX00009; Proteintech), Sirt2 (1:10000; 19655‐1‐AP; Proteintech), Sirt3 (1:2000; HA722251; HUABIO), Sirt4 (1:3000; DF8476; Affinity), Sirt5 (1:8000; 15122‐1‐AP; Proteintech), Sirt6 (1:1500; 13572‐1‐AP; Proteintech), Sirt7 (1:2000; 12994‐1‐AP; Proteintech), ubiquitin (1:1000; sc‐8017; Santa Cruz Biotechnology), Actyllysine (1:2000; PTM‐105RM; PTMBIO), GAPDH (1:1000; AG109; Beyotime), Actin (1:1000; AA128; Beyotime) overnight at 4°C. Following three TBS‐Tween washes, membranes were incubated with appropriate secondary antibodies for 1 h at room temperature. Protein bands were visualized using chemiluminescent peroxidase substrate (PK10003; Proteintech).

### 4.5 ASC Speck

Following stimulation, cells were fixed with 4% paraformaldehyde and processed for immunofluorescence staining using standard procedures. Briefly, samples were incubated with an anti‐ASC primary antibody (1:500; 67824S, Cell Signaling Technology), followed by an appropriate anti‐rabbit secondary antibody. Nuclei were counterstained with DAPI (1:1000; C1002, Beyotime) as per the manufacturers’ protocols. Fluorescent images were acquired using a Leica confocal microscope (Wetzlar, Germany) and subsequently analyzed.

### 4.6 ASC Oligomerization

Following stimulation, the cells were washed twice with PBS and subsequently lysed in an ice‐cold buffer (pH 7.6) composed of 50 mm Tris‐HCl, 0.5% Triton X‐100, 0.1 mm PMSF, and a protease inhibitor cocktail. The lysates were then collected by scraping and centrifuged at 3000 g for 10 min. After removing the supernatant, the resulting pellets were washed twice with PBS. Next, the pellets were resuspended and incubated with 2 mm disuccinimidyl suberate (DSS; A39267, Thermo Fisher) for 30 min at room temperature to facilitate cross‐linking. Finally, the cross‐linked pellets were collected and prepared for immunoblot analysis.

### 4.7 Osteoclasts Differentiation

Bone marrow cells were flushed from bilateral tibiae and femurs and culture in α‐MEM medium. The next day, non‐adherent cells were collected and seeded into 6‐well or 24‐well plates with α‐MEM culture medium containing 10% fetal bovine serum and 40ng/mL M‐CSF. Three days later, the culture medium was added with RANKL (50 ng/mL) and replaced every 3 days. Mature osteoclasts were obtained after 5‐6 days and subjected to further experiments.

### 4.8 Bone Marrow Derived Mesenchymal Stem Cells Isolation and Osteoblasts Differentiation

Bone marrow derived mesenchymal stem cells (BMSCs) were isolated as previously described. For osteoblast differentiation, BMSCs were cultured in α‐MEM containing 10% fetal bovine serum, 10 mm β‐glycerol phosphate, 50 mm ascorbate‐2‐phosphate, and 0.1 mm dexamethasone for 21 days. Differentiated osteoblasts were subjected for further experiments.

### 4.9 Immunofluorescence

Cells were seeded at 1 × 10^6^ cells/well in 24‐well plates containing 12‐mm coverslips (Biosharp, Hefei, China) and cultured in osteoclast differentiation medium at 37°C under 5.5% CO_2_ until full differentiation. After fixation, F‐actin rings were visualized using Actin‐Tracker Red‐Rhodamine (C2207S, Beyotime) following the manufacturer's protocol. Additionally, immunofluorescence staining was performed using primary antibodies against TRAF6 (1:200; sc‐8409; Santa Cruz Biotechnology) and NFATc‐1 (1:20; ab25916; Abcam), with nuclei counterstained using DAPI.

### 4.10 Calcein Double Staining

To assess bone formation dynamics, a calcein‐double labeling approach was employed. Mice received subcutaneous injections of 0.1% calcein (C0875, Sigma–Aldrich) dissolved in phosphate‐buffered saline at a dosage of 10 mg/kg body weight. The injections were administered 7 days and 1 day prior to euthanasia. Images were captured by the fluorescence microscopy. Quantitative evaluation of trabecular bone formation was conducted by analyzing five randomized visual fields within the distal femur.

### 4.11 Bioinformatics Analysis of Bulk RNA‐seq

Transcriptional studies comparing macrophages derived from young and aged mice (data sets GEO GSE98249) were utilized. To identify differentially expressed genes (DEGs) between young and aged macrophages, transcript abundance was quantified using RSEM and normalized by the transcripts per million (TPM) method. Gene expression differences were statistically evaluated with either DESeq2 or DEGseq, with significance thresholds set at |log2FC|≥1 combined with FDR < 0.05 for DESeq2 or FDR < 0.001 for DEGseq. Subsequently, functional annotation of the identified DEGs was conducted through Gene Ontology (GO) and KEGG pathway enrichment analyses. These analyses were implemented using Goatools and Python scipy packages, respectively, with statistically significant terms defined as those achieving a Bonferroni‐adjusted *p*‐value < 0.05 relative to the complete transcriptome background.

### 4.12 qRT‐PCR Analysis

Total RNA was isolated with TRIzol reagent following the manufacturer's protocol. For cDNA synthesis, 1 µg of RNA from each sample was reverse transcribed using the RevertAid First Strand cDNA Synthesis Kit (Thermo Fisher Scientific, Waltham, MA, USA). Quantitative PCR amplification was then performed with iTaq Universal SYBR Green Supermix (Bio‐Rad, Hercules, CA, USA). Primer sequences are provided in Table S1.

### 4.13 Transfection and Immunoprecipitation

HEK293T cells were transfected with different constructs by polyethylenimine (40816ES03, Yeasen Biotechnology). 24h later, total cell lysates were collected and immunoprecipitated with anti‐Flag antibody (1:50; 14793S, Cell Signaling Technology) and assessed by western blot. All constructs were purchased from MIAOLING biology. For immunoprecipitation, total cell lysates were collected and immunoprecipitated by the primary antibodies against NLRP3 (AG‐20B‐0014‐C100, 1:100, AdipoGen). Then, the experimental procedure was performed according to the immunoprecipitation Kit with Protein A+G Magnetic Beads (P2179S, Beyotime). Finally, samples were separated by SDS‐PAGE and blotted onto a PVDF membrane (Millipore). Next, membranes were incubated with the primary antibodies against Ubiquitin (SC‐8017, Santa), Ac‐lysine (SC‐81623, Santa), and the second antibodies. The band was visualized by a ultrasensitive chemiluminescence detection kit (PK10003, Proteintech).

### 4.14 MicroCT Analysis

Micro‐CT analysis was performed as previously described. Briefly, Femur and lumbar were collected and scanned by a Micro‐CT device (SkyScan 1275, Aartselaar, Belgium). The scan parameters were set as follows: 500 µA (current), 50 kV (voltage), and 13 µm (per layer). The bone parameters and 3D reconstruction were performed with CTAN and mimics software. The parameters of BV/TV (Bone volume/Tissue volume), Tb.Th (Trabecular thickness), Tb.N (Trabecular number), Tb.Sp (Trabecular separation), and BMD (Bone mineral density) were calculated by the software of CTAN.

### 4.15 Apoptotic Bodies Induction and Isolation

ABs (Apoptotic bodies) induction and isolation were performed as previously described. Briefly, BMSCs were treated with staurosporine (5 µm, abs810006, Absin) for 12h. The supernatants were collected and centrifuged at 300 g for 10 min to eliminate cell debris. The remaining supernatants were further centrifuged at 3000 × g for 10 min to isolated ABs and then resuspended in PBS. In vitro experiments, BMDMs were incubated with ABs (30 µg/mL) for 12 h and then subjected to further experiments. In vivo experiments, aged male mice (20 months) were intravenously injected with PBS or different ABs (100 µg dissolved in 100 µL PBS) twice a week for 8 weeks. The morphological characteristics of ABs were examined using transmission electron microscopy (TEM). Briefly, the ABs were fixed in 1% glutaraldehyde for 30 min before being deposited onto formvar‐carbon‐coated copper grids and allowed to adhere for 20 min. Following a washing step, the samples were contrast‐enhanced with 2.5% uranyl acetate for 2 min. Imaging was performed employing a JEM‐1200EX TEM system (Hitachi, HT‐7700, Japan). The zeta potential and size distribution of ABs were measured by Zetasizer (Nano‐ZS90, Malvern). The protein concentration of ABs were determined using a BCA assay kit (Beyotime, Shanghai, China). Markers of ABs were examined by Western Blot with primary bodies H3 (1:1000; sc‐517576; Santa Cruz Biotechnology), H2B (1:1000; sc‐515808; Santa Cruz Biotechnology), C3B (1:1000; sc‐28294; Santa Cruz Biotechnology), and C1QC (1:1000; sc‐365301; Santa Cruz Biotechnology).

### 4.16 ELISA

The supernatants were collected for the detection of IL‐1β and IL‐18 by using the ELISA kits (EK201BHS and EK218, Multi Sciences) according to the instructions of the manufacturer. The level of serum OCN was measured by an ELISA kit (KE1428, Immunoway).

### 4.17 Histological Analysis

Femur and lumbar of mice from each group were collected and fixed in 4% paraformaldehyde. Then, these samples were decalcified for a month and embedded into the paraffin. Samples were cut to 6 µm slices and then performed with Hematoxylin‐Eosin (HE; DH0006; LEAGENE), Tartrate‐resistant acid phosphatase (TRAP; G1492; Solarbio), and Toluidine Blue (TB; DB0057; LEAGENE) staining for the evaluation of fat accumulation, bone erosion, and new bone formation.

### 4.18 Statistical Analysis

Quantitative data were expressed as mean ± SD. Two‐group comparisons were analyzed using a two‐tailed Student's t‐test, while multi‐group comparisons employed a one‐way analysis of variance (ANOVA) and post‐hoc (Tukey) test. All in vitro experiments included ≥ 3 biological replicates, with representative results displayed. *p* < 0.05 (^*^), *p* < 0.01 (^**^), *p* < 0.001 (^***^) was considered statistically significant.

## Author Contributions

**Wenguo Cui**: **Ming Cai**: and **Yanglin Wu**: designed the research. **Yanglin Wu**: **Shifeng Ling**: and **Jiayi Mao**: performed the experiments. **Hongyi Wang**: and **Bo Wang**: analyzed the data. **Yanglin Wu**: wrote the paper.

## Funding

Our study was supported by the National Natural Science Foundation of China (82272176).

## Conflicts of Interest

The authors declare no conflicts of interest.

## Supporting information




**Supporting File**: advs74587‐sup‐0001‐SuppMat.docx

## Data Availability

The data that support the findings of this study are available from the corresponding author upon reasonable request.
