# Supplementary material for: Sirt3 Genetically Engineered Apoptotic Bodies Alleviate Skeletal Aging by Limiting Aggravated NLRP3 Inflammasome Activation of Senescent Macrophages
Source: Adv Sci (Weinh). 2026 Feb 27;13(26):e17554. doi: 10.1002/advs.202517554 (PMC13159149; doi:10.1002/advs.202517554)
Supplement: Supplementary file 1 — Supporting File: advs74587‐sup‐0001‐SuppMat.docx [file ADVS-13-e17554-s001.docx]

**Supporting Information for**

Sirt3 genetically engineered apoptotic bodies alleviate skeletal aging by limiting aggravated NLRP3 inflammasome activation of senescent macrophages

*Yanglin Wu^1#^, Shifeng Ling^2#^, Jiayi Mao^3#^, Hongyi Wang^2^, Bo Wang^4^, Zhenjia Che^1^, Wenguo Cui^2*^, Ming Cai^1*^*

**This file includes:**

Supplementary Text

Figs. S1 to S9

Tables S1 to S3

Supplementary Text

**
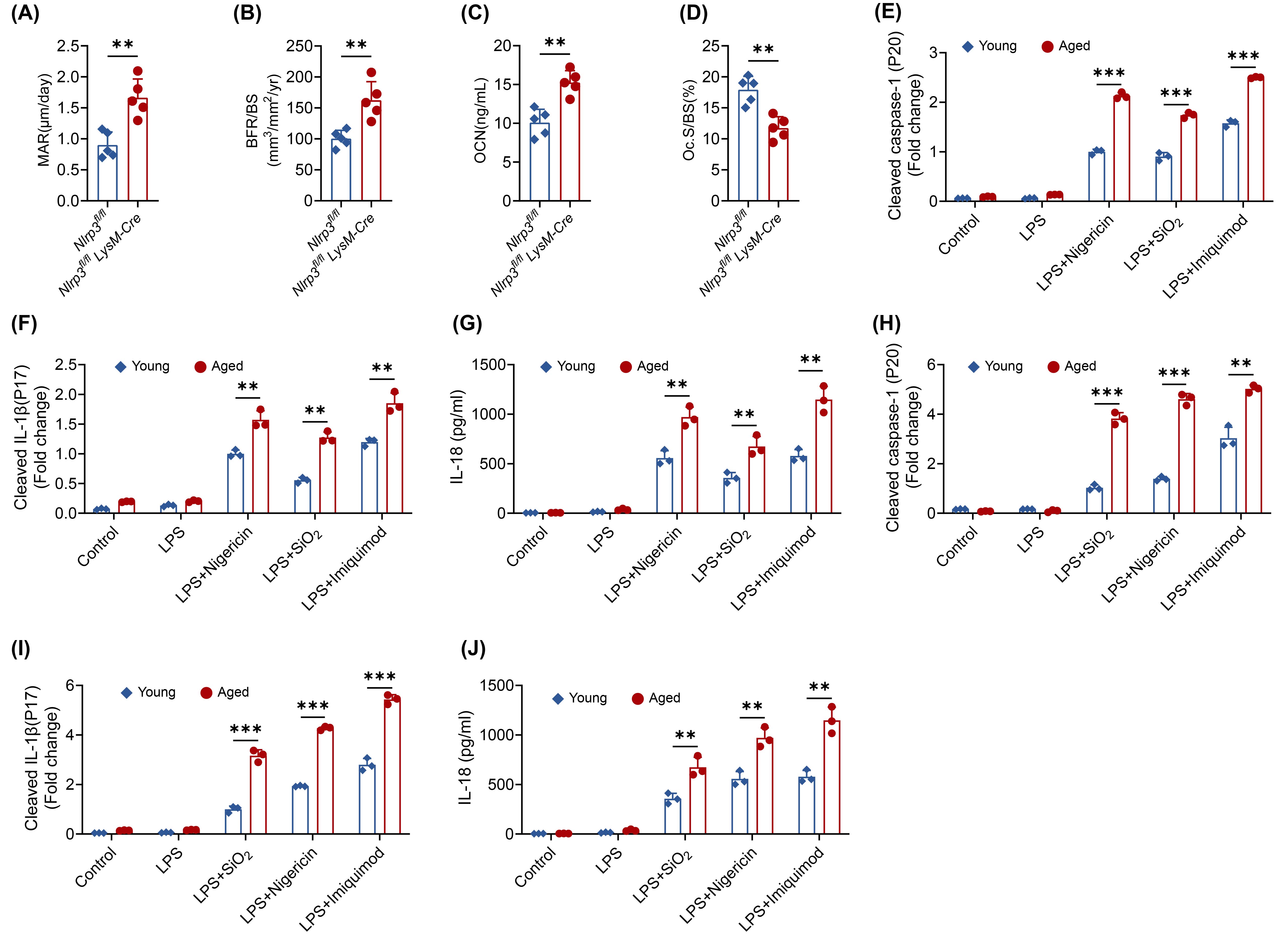
**

**Fig. S1. NLRP3 inflammasome activation was aggravated in aged macrophages.** (A) Quantitative analysis of mineral apposition rate (n=5). (B) Quantitative analysis of bone formation rate (n=5). (C) Serum level OCN in *Nlrp3^fl/fl^*, *Nlrp3^fl/fl^LysM-Cre* mice. (n=5). (D) Quantification of osteoclasts surface per bone surface (Oc.S/BS). (E) Quantification analysis of cleaved caspase-1 (P20) in macrophages derived from young and aged mice. (F) Quantification analysis of cleaved IL-1β (P17) in macrophages derived from young and aged mice. (G) The secretion of IL-18 analyzed by ELISA in macrophages derived from young and aged mice (n=3). (H) Quantification analysis of cleaved caspase-1 (P20) in PBMCs derived from young and aged people. (I) Quantification analysis of cleaved IL-1β (P17) in macrophages derived from PBMCs derived from young and aged people. (J) The secretion of IL-18 analyzed by ELISA in macrophages derived from PBMCs derived from young and aged people (n=3). Data were shown as means±SD. Each point in scatter plots represented one individual. ns (no significance), ***P* < 0.01, and ****P* < 0.001, by two tailed *t* test.


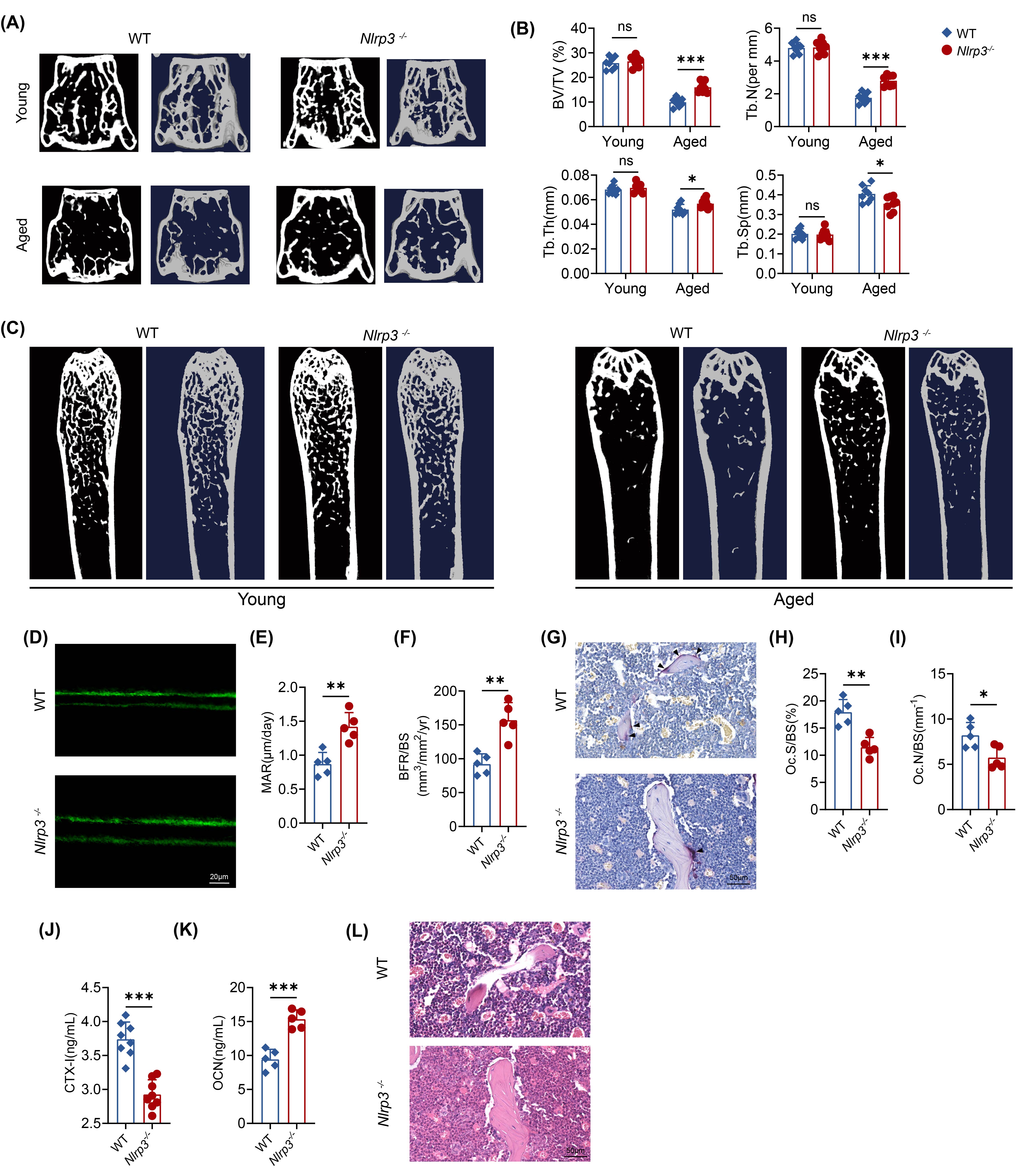


**Fig. S2. Knockout of NLRP3 alleviated age-related osteoporosis.** (A) Representative microCT images of vertebra. (B) Quantitative analysis of femur trabecular bone volume (BV/TV; %), trabecular separation (Tb.Sp; mm), trabecular thickness (Tb.Th; mm), and trabecular bone number (Tb.N; 1/mm) (n=8). (C) Representative microCT images of femur. (D) Representative image of calcein double labeling of femur trabecular bones. Scale bar: 20 μm. (E) Quantitative analysis of mineral apposition rate (n=5). (F) Quantitative analysis of bone formation rate (n=5). (G) TRAP staining of distal femur. Scale bar: 50 μm. Black arrows indicate osteoclasts. (H) Quantitative analysis of osteoclasts surface per bone surface (Oc.S/BS). (I) Quantitative analysis of osteoclasts number per bone surface (Oc.N/BS). (J) Serum level of CTX in WT and *Nlrp3* KO mice (n=8). (K) Serum level of OCN in WT and *Nlrp3* KO mice (n=8). (L) HE staining of distal femur. Scale bar: 50 μm. Data were shown as means±SD. Each point in scatter plots represented one individual. ns (no significance), **P* < 0.05, ***P* < 0.01, and ****P* < 0.001, by two tailed *t* test.





**Fig. S3. Ablation of Sirt2 or Sirt3 aggravated the activation of NLRP3 inflammasome.** (A) PCA plot of young and aged macrophages. (B) Upregulated GO categories in aged macrophages compared with young macrophages. (C) Upregulated KEGG categories in aged macrophages compared with young macrophages. (D-E) The mRNA expression level of autophagy-related genes in young and aged macrophages. (F) Representative confocal images of young and old macrophages transfected with mCherry-GFP-LC3 construct. (G) Quantification analysis of autolysosomes and autophagosomes in young and senescent macrophages (n=3). (H) Expression of mTOR and AMPK pathway-related genes in macrophages. (I) Expression of HDAC family genes in macrophages. (J) BMDMs were left untreated or treated with RGFP966 or Salvianolic acid B respectively and then activated the NLRP3 inflammasome. The supernatants were subjected to the IL-1β secretion by ELISA (n=3). (K) Expression of Foxo family genes in macrophages. (L) Expression of *Nfe2l2*, *Parp1*, *Tert*, *Foxp1*, *Kl*, *Tet2* in macrophages. (M) Quantitative analysis of caspase-1 and IL-1β of BMDMs treated with none (Control), AGK2, or 3-TYP (n=3). (N) Quantitative analysis of caspase-1 and IL-1β of BMDMs from *Sirt2^fl/fl^* or *Sirt2^fl/fl^* LysM-Cre mice (n=3). (O) Quantitative analysis of caspase-1 and IL-1β of BMDMs from *Sirt3^fl/fl^* or *Sirt3^fl/fl^* LysM-Cre mice (n=3). (P-Q) The mRNA level of Sirt2 (P) and IL-1β release (Q) in macrophages treated as indicated (n=3). (R-S) The mRNA level of Sirt3 (R) and IL-1β release (S) in macrophages treated as indicated (n=3). Data were shown as means±SD. Each point in scatter plots represented one individual. ns (no significance), **P* < 0.05, ***P* < 0.01, and ****P* < 0.001. Two tailed *t* test for (D), (E), (H), (I), (K), (L), (N), and (O). One-way ANOVA followed by Tukey’s post hoc analysis for (J), (M), (P), (Q), (R), and (S).


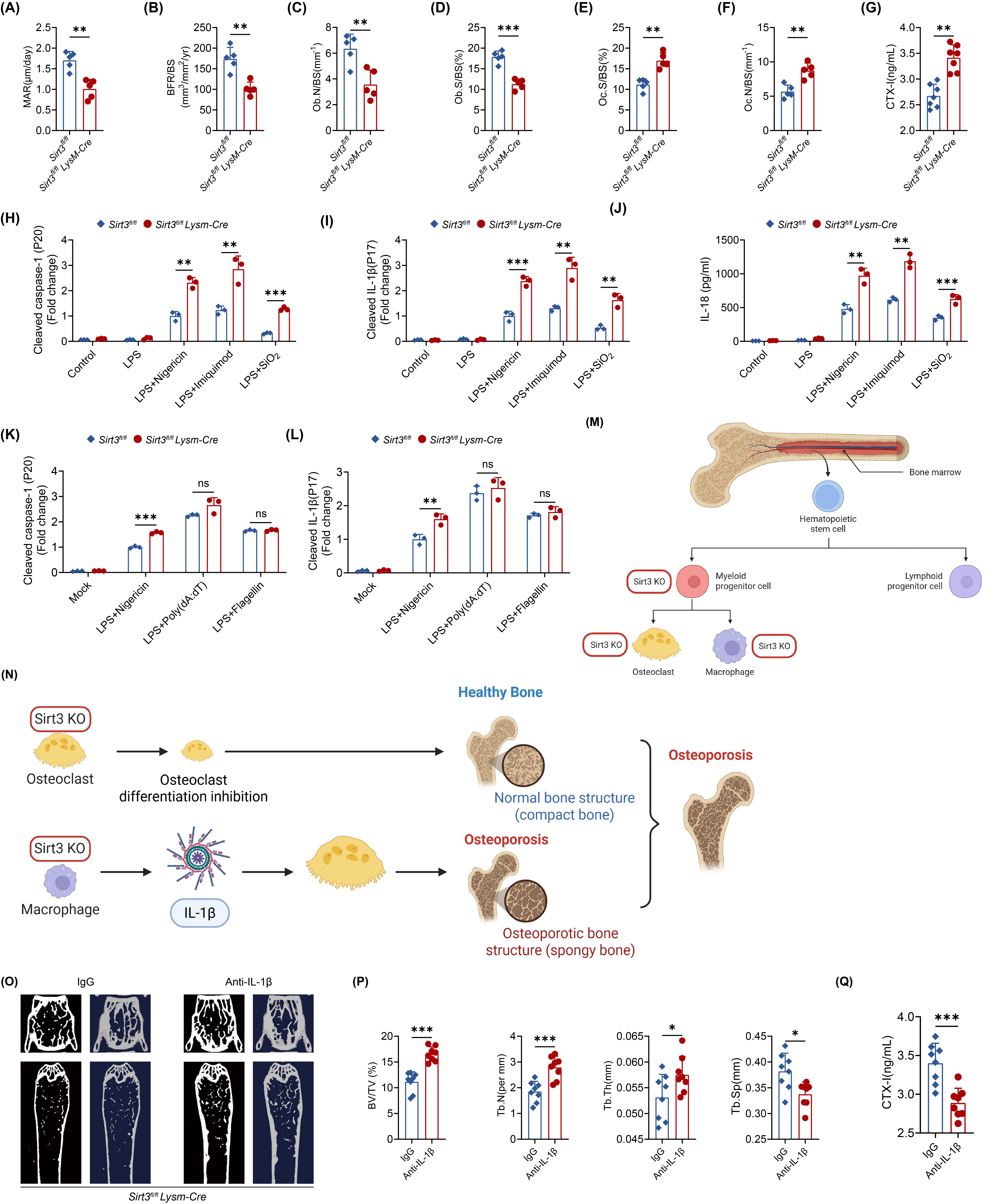


**Fig. S4. NLRP3 inflammasome activation was aggravated in Sirt3 deficient macrophages.** (A) Quantitative analysis of mineral apposition rate (n=5). (B) Quantitative analysis of bone formation rate (n=5). (C) Quantification of osteoblasts number per bone surface (Ob.N/BS). (D) Quantification of osteoblasts surface per bone surface (Ob.S/BS). (E) Quantification of osteoclasts surface per bone surface (Oc.S/BS). (F) Quantification of osteoclasts number per bone surface (Oc.N/BS). (G) Serum level CTX-I in *Sirt3^fl/fl^*, *Sirt3^fl/fl^ LysM-Cre* mice (n=5). (H) Quantification analysis of cleaved caspase-1 (P20) in macrophages derived from *Sirt3^fl/fl^*, *Sirt3^fl/fl^ LysM-Cre* mice. (I) Quantification analysis of cleaved IL-1β (P17) in macrophages derived from *Sirt3^fl/fl^*, *Sirt3^fl/fl^ LysM-Cre* mice. (J) The secretion of IL-18 analyzed by ELISA in macrophages derived from *Sirt3^fl/fl^*, *Sirt3^fl/fl^ LysM-Cre* mice (n=3). (H) Quantification analysis of cleaved caspase-1 (P20) in macrophages derived from *Sirt3^fl/fl^*, *Sirt3^fl/fl^ LysM-Cre* mice. (K) Quantification analysis of cleaved IL-1β (P17) in macrophages derived from *Sirt3^fl/fl^*, *Sirt3^fl/fl^ LysM-Cre* mice. (L) The secretion of IL-18 analyzed by ELISA in macrophages derived from *Sirt3^fl/fl^*, *Sirt3^fl/fl^ LysM-Cre* mice (n=3). (M) Schematic diagram of macrophage and osteoclast differentiation. (N) Schematic diagram of the effect of Sirt3 deficiency in osteoclasts and macrophages on bone mass (O) 3D-reconstructed MicroCT images of femur and vertebra. (P) Quantitative femoral trabecular parameters: BV/TV (%); Tb.Sp (mm); Tb.Th (mm); Tb.N (1/mm). n=8. (Q) Serum level of CTX in IgG or Anti-IL-1β treated *Sirt3^fl/fl^LysM-Cre* mice. n=8. Data were shown as means±SD. Each point in scatter plots represented one individual. ns (no significance), ***P* < 0.01, and ****P* < 0.001, by two tailed *t* test.


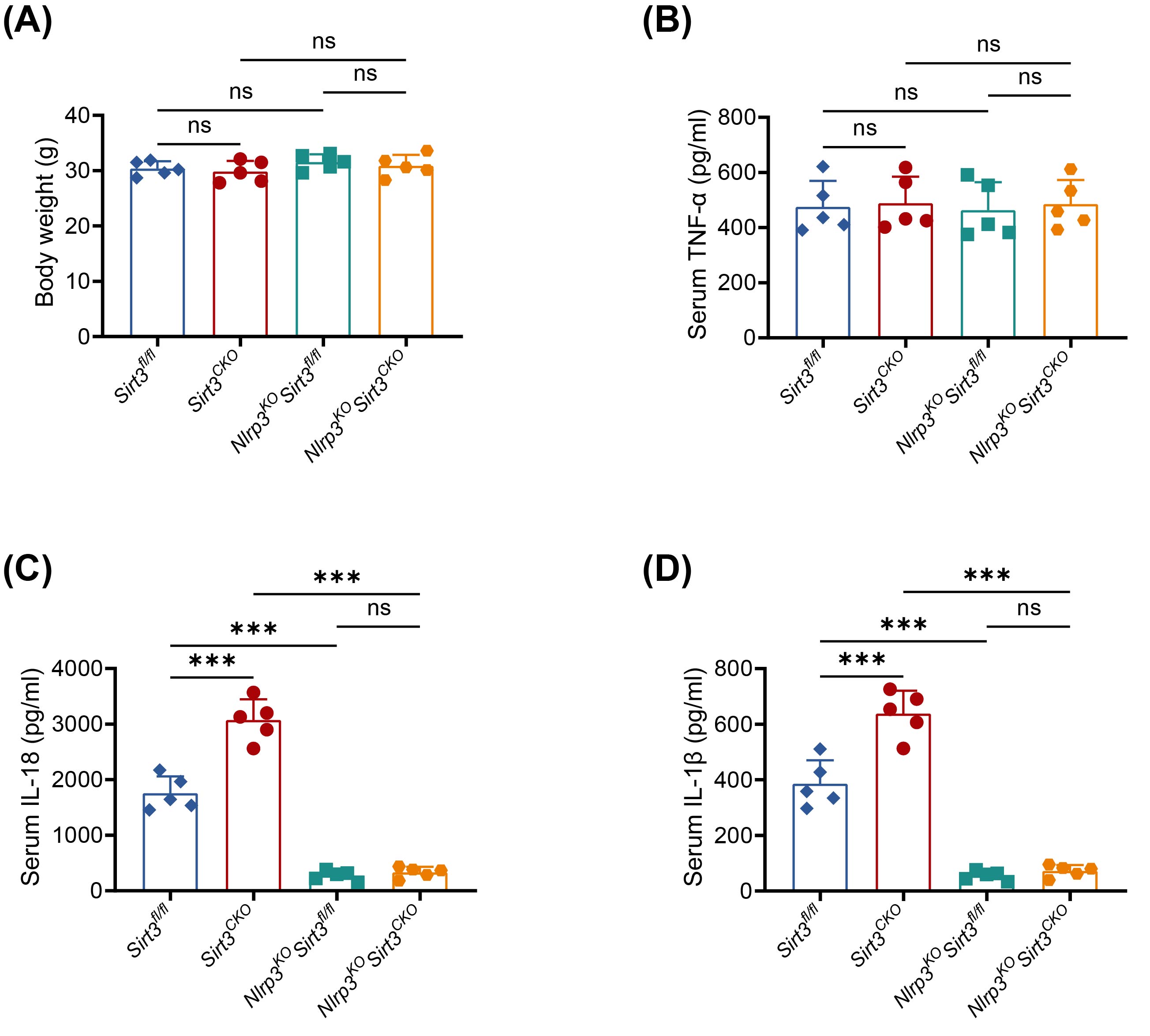


**Fig. S5. NLRP3 knockout reduced the secretion of IL-1β and IL-18 promoted by Sirt3 deficiency.** (A) Body weight of *Sirt3^fl/fl^*, *Sirt3^CKO^*, *Nlrp3^KO^ Sirt3^fl/fl^*, and *Nlrp3^KO^ Sirt3^CKO^* mice. (n=5). (B-D) Serum level of IL-1β (B), IL-18 (C), and TNF-α (D) in *Sirt3^fl/fl^*, *Sirt3^CKO^*, *Nlrp3^KO^ Sirt3^fl/fl^*, and *Nlrp3^KO^ Sirt3^CKO^* mice intraperitoneally injected with LPS (25 mg/kg body weight) for 4h (n=5). Data were shown as means±SD. Each point in scatter plots represented one individual. ns (no significance), and ****P* < 0.001, by One-way ANOVA followed by Tukey’s post hoc analysis.


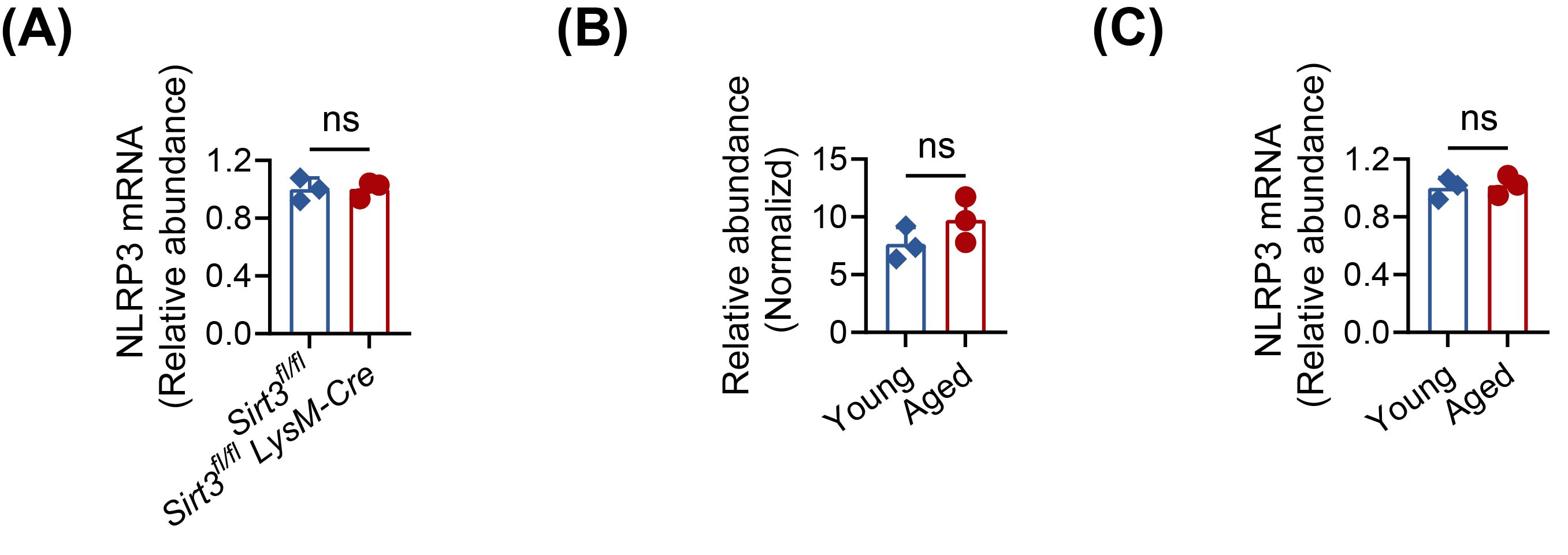


**Fig. S6.** **Aging did not lead to a significant increase in NLRP3 mRNA level.** (A) Expression of NLRP3 in macrophages derived from Sirt3^fl/fl^ or Sirt3^fl/fl^ LysM-Cre micre (n=3). (B) Expression of NLRP3 in young and aged macrophages from GSE98249 (n=3). (C) Expression of NLRP3 in macrophages derived from young and aged mice (n=3). Data were shown as means±SD. Each point in scatter plots represented one individual. ns (no significance), by two tailed *t* test.

**
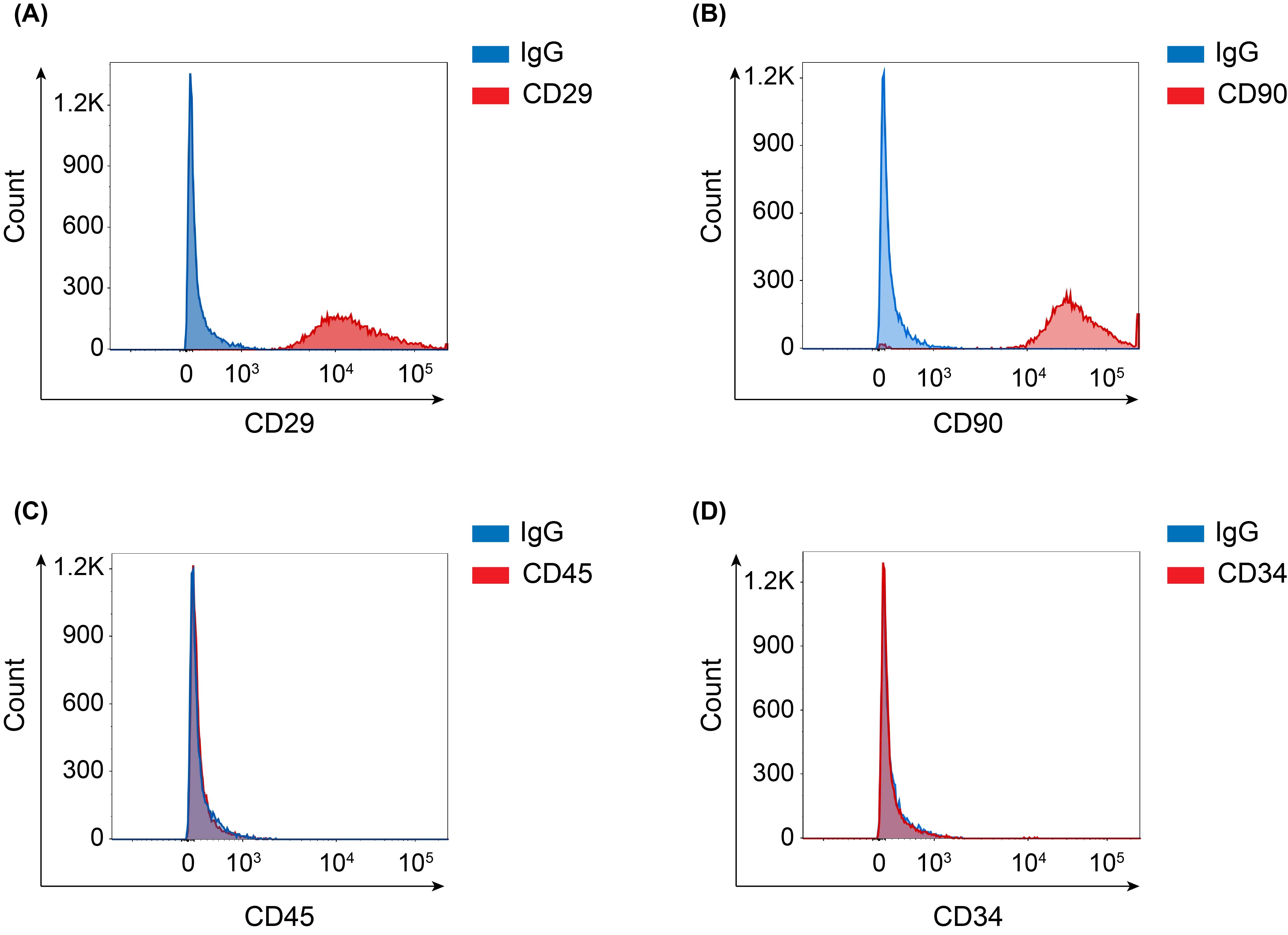
**

**Fig. S7. Immunophenotypes of bone marrow mesenchymal stem cells (BMSCs).** (A) Flow cytometry analysis of CD29 expression in BMSCs. (B) Flow cytometry analysis of CD90 expression in BMSCs. (C) Flow cytometry analysis of CD45 expression in BMSCs. (D) Flow cytometry analysis of CD34 expression in BMSCs.


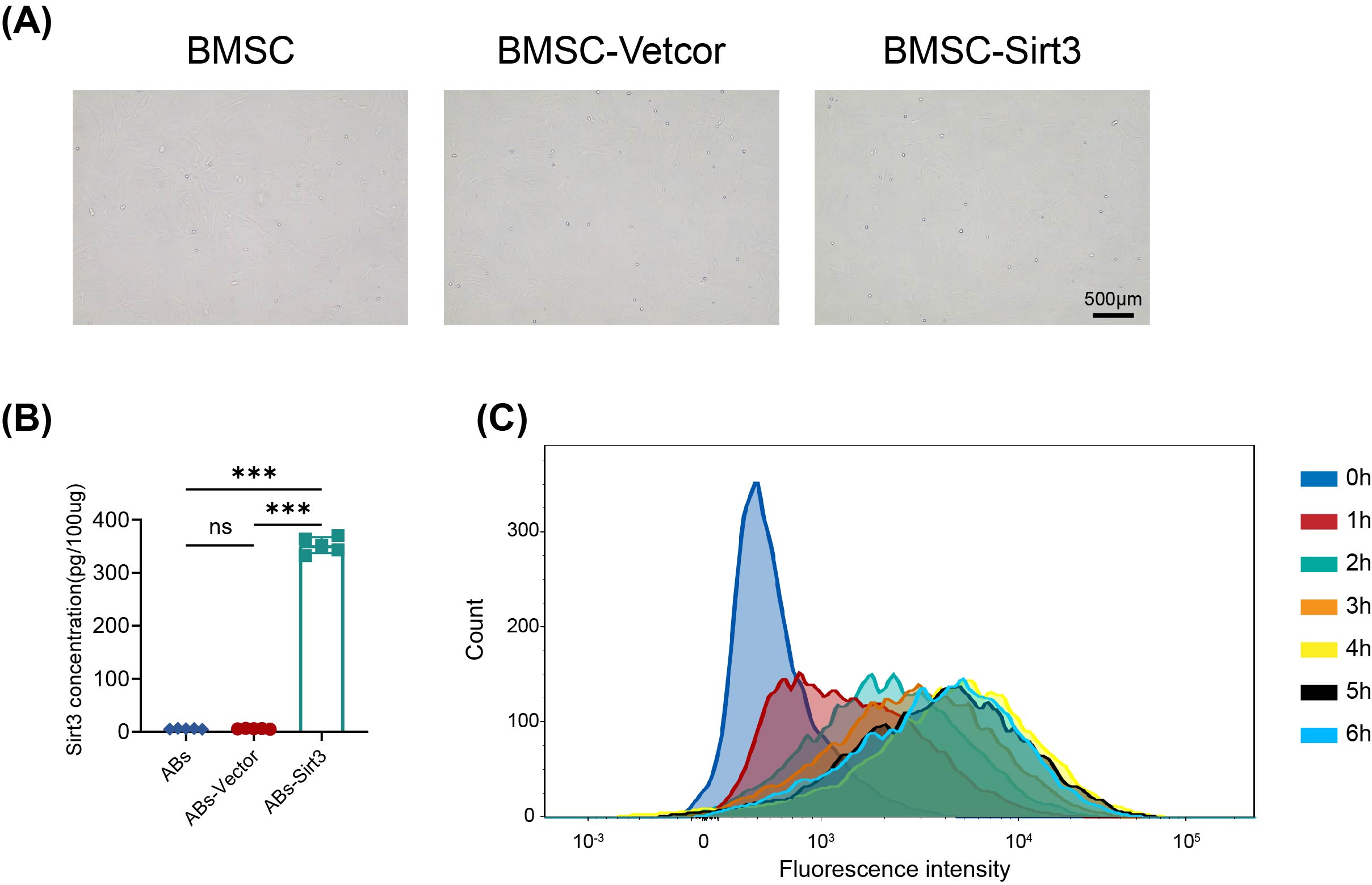


**Fig. S8.** Characteristics of apoptotic bodies. (A) Representative images of BMSCs with nontransfection, or lentivirus transfection. (B) The protein concentration of Sirt3 in apoptotic bodies. (C) Fluorescently labeled apoptotic bodies were co-cultured with macrophages and analyzed by flow cytometry at 0, 1, 2, 3, 4, 5, and 6 hours, respectively. Data were shown as means±SD. Each point in scatter plots represented one individual. ns (no significance), and ****P* < 0.001, by One-way ANOVA followed by Tukey’s post hoc analysis.

**
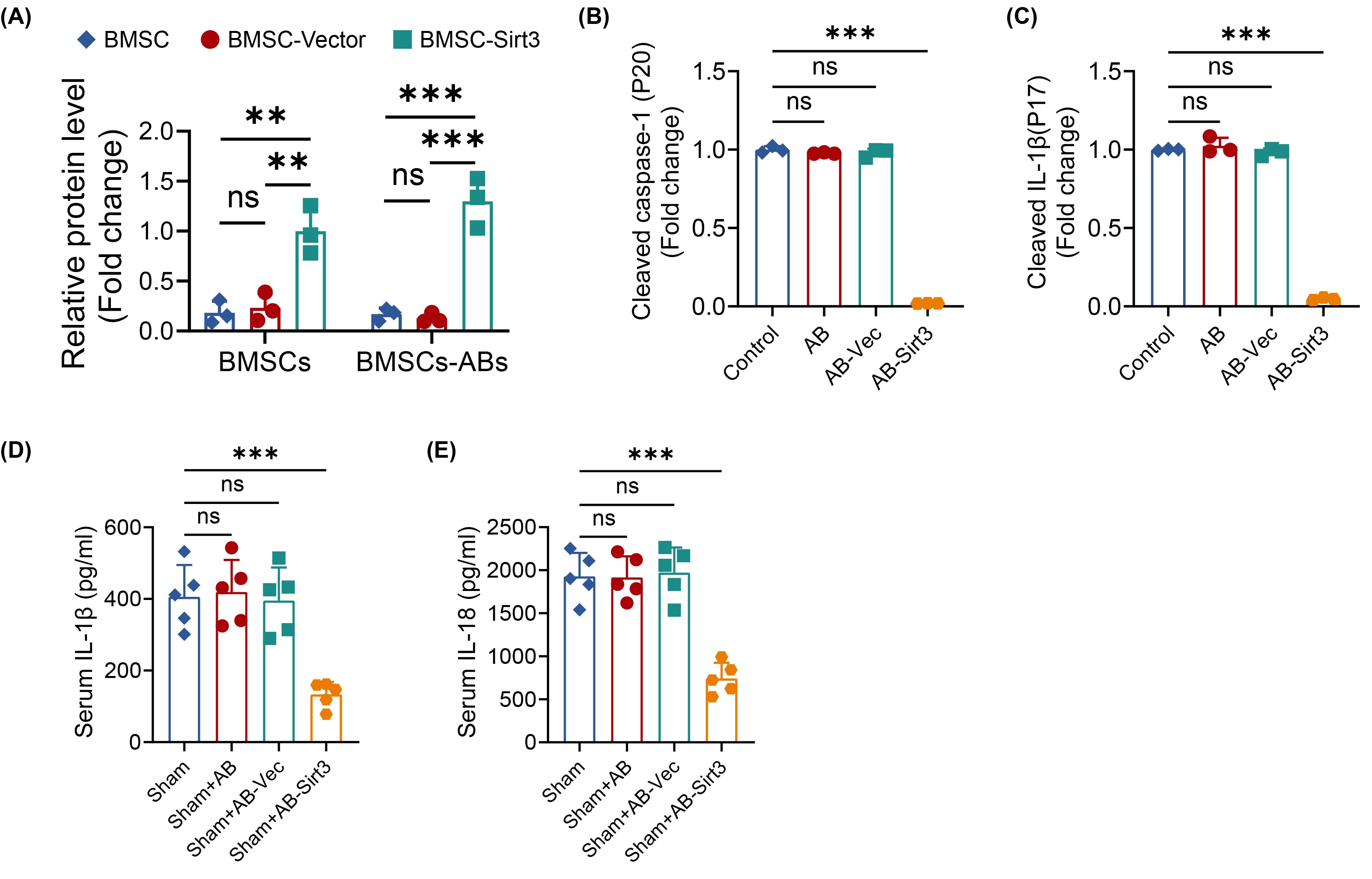
**

**Fig. S9. Sirt3-enriched apoptotic bodies deactivated the NLRP3 inflammasome.** (A-B) Quantitative analysis of caspase-1 (A) and IL-1β (B) (n=3). (C-D) The serum level of IL-1β (C) and IL-18 (D) in mice treated as indicated (n=5). Data were shown as means±SD. Each point in scatter plots represented one individual. ns (no significance), ***P* < 0.001, and ****P* < 0.001, by One-way ANOVA followed by Tukey’s post hoc analysis.

| **Gene** | **Forward** | **Reverse** |
| --- | --- | --- |
| ***NFATc-1*** | 5´GGTGCCTTTTGCGAGCAGTATC3´ | 5´CGTATGGACCAGAATGTGACGG3´ |
| ***Ocstamp*** | 5´TTGCTCCTGTCCTACAGTGC3´ | 5´GCCCTCAGTAACACAGCTCA3´ |
| ***Acp5*** | 5´CGACCATTGTTAGCCACATACG3´ | 5´TCGTCCTGAAGATACTGCAGGTT3´ |
| ***Ctsk*** | 5´AGGGCCAACTCAAGAAGAAAACT3´ | 5´TGCCATAGCCCACCACCAACACT3´ |
| ***Mmp9*** | 5´GCTGACTACGATAAGGACGGCA3´ | 5´ TAGTGGTGCAGGCAGAGTAGGA3´ |
| ***VEGF*** | 5´CTGCTGTAACGATGAAGCCCTG3´ | 5´GCTGTAGGAAGCTCATCTCTCC3´ |
| ***IL-6*** | 5´TACCACTTCACAAGTCGGAGGC3´ | 5´CTGCAAGTGCATCATCGTTGTTC3´ |
| ***Oct-4*** | 5´CAGCAGATCACTCACATCGCCA3´ | 5´GCCTCATACTCTTCTCGTTGGG3´ |
| ***Rex-1*** | 5´GAGACTGAGGAAGATGGCTTCC3´ | 5´CTGGCGAGAAAGGTTTTGCTCC3´ |
| ***Sirt1*** | 5´GGAGCAGATTAGTAAGCGGCTTG3´ | 5´GTTACTGCCACAGGAACTAGAGG3´ |
| ***Sirt2*** | 5´CGAAGGAGTGACACGCTACATG3´ | 5´GGTGGTACTTCTCCAGGTTTGC3´ |
| ***Sirt3*** | 5´GCTACATGCACGGTCTGTCGAA3´ | 5´CAATGTCGGGTTTCACAACGCC3´ |
| ***Sirt4*** | 5´TCCGCTGCTCAAGATCCCTA3´ | 5´CTTTCCTTAGGGGGCAGCTC3´ |
| ***Sirt5*** | 5´ATCGCAAGGCTGGCACCAAGAA3´ | 5´CTAAAGCTGGGCAGATCGGACT3´ |
| ***Sirt6*** | 5´CAGTACGTCAGAGACACGGTTG3´ | 5´GTCCAGAATGGTGTCTCTCAGC3´ |
| ***Sirt7*** | 5´CTGGAGATTCCTGTCTACAACCG3´ | 5´AGTGACTTCCTACTGTGGCTGC3´ |
| ***Runx2*** | 5´CCTTCAAGGTTGTAGCCCTC3´ | 5´GGAGTAGTTCTCATCATTCCCG3´ |
| ***Ocn*** | 5´TTGAACTGTTTGTTTTGGACCC3´ | 5´CCAACAGACACCAGTTGTAAAG3´ |
| ***Sp7*** | 5´TCGTCTGACTGCCTGCCTAGTG3´ | 5´CTGCGTGGATGCCTGCCTTG3´ |
| ***GAPDH*** | 5´GGTTGTCTCCTGCGACTTCA3´ | 5´TGGTCCAGGGTTTCTTACTCC3´ |

**Table. S1.** Primer sequences in qRT-PCR analysis.

| **Age**  **(year)** | **Sex** | **Height**  **(m)** | **Weight**  **(kg)** | **BMI** | **Health status** |
| --- | --- | --- | --- | --- | --- |
| 22 | male | 1.72 | 72 | 24.34 | health |
| 25 | male | 1.75 | 68 | 22.20 | health |
| 27 | male | 1.78 | 69 | 21.78 | health |
| 66 | male | 1.74 | 71 | 23.45 | health |
| 72 | male | 1.71 | 70 | 23.04 | health |
| 70 | male | 1.77 | 75 | 23.94 | health |

**Table. S2.** Demographics and clinical data

|  | **Young(3)** | **Old(3)** | **p** |
| --- | --- | --- | --- |
| Age | 24.67±2.52 | 69.33±3.06 | 0.00004 |
| BMI | 23.49±1.48 | 23.48±0.45 | 0.992 |

**Table. S3.** Demographics data of young and old healthy donors
